# Dose–Response Relationships for Lung Tumor and Pleural Mesothelioma Induction by Repetitive Intratracheal Instillation of a Multiwalled Carbon Nanotube, MWCNT-7, in Rats: Comparison with Inhalation Carcinogenicity Based on Lung Burden

**DOI:** 10.3390/nano16161039

**Published:** 2026-08-20

**Authors:** Ai Maeno, Motoki Hojo, Yoshimitsu Sakamoto, Yukio Yamamoto, Kiyomi Ikushima, Kai Igarashi, Satoshi Yokota, Yuhji Taquahashi, Norihiro Kobayashi, Hiroyuki Tsuda, Aya Naiki-Ito, Takamasa Numano, Jun Kanno, Akihiko Hirose, Akiko Inomata, Junichi Kamiie, Dai Nakae

**Affiliations:** 1 Department of Pharmaceutical and Environment Health, Tokyo Metropolitan Institute of Public Health, 3-24-1 Hyakunincho, Shinjuku-Ku, Tokyo 169-0073, Japan; 2 Laboratory of Veterinary Pathology, Graduate School of Veterinary Medicine, Azabu University, 1-17-71 Fuchinobe, Chuo-Ku, Sagamihara 252-0206, Japan; 3 Division of Cellular and Molecular Toxicology, Center for Biological Safety and Research, National Institute of Health Sciences, 3-25-26 Tono-Machi, Kawasaki-Ku, Kawasaki 210-9501, Japan; 4 Division of Environmental Chemistry, National Institute of Health Sciences, 3-25-26 Tono-Machi, Kawasaki-Ku, Kawasaki 210-9501, Japan; 5 Nanotoxicology Project, Graduate School of Medical Sciences, Nagoya City University, 1 Aza Kawasumi, Mizuho-Cho, Mizuho-Ku, Nagoya 467-8601, Japan; 6 Department of Experimental Pathology and Tumor Biology, Graduate School of Medical Sciences, Nagoya City University, 1 Aza Kawasumi, Mizuho-Cho, Mizuho-Ku, Nagoya 467-8601, Japan; 7 National Institute of Occupational Safety and Health, Japan Organization of Occupational Health and Safety, 2-26-1 Muraokahigashi, Fujisawa 251-0012, Japan; 8 Chemicals Assessment and Research Center, Chemicals Evaluation and Research Institute, Japan, 1-4-25 Koraku, Bunkyo-Ku, Tokyo 112-0004, Japan; 9 Department of Medical Sports, Faculty of Health Care and Medical Sports, Teikyo Heisei University, 4-1 Uruido-Minami, Ichihara 290-0193, Japan

**Keywords:** MWCNT, carcinogenicity, intratracheal instillation, lung tumors, pleural mesothelioma, lung burden, dose–response relationship, NOAEL

## Abstract

The multiwalled carbon nanotube, MWCNT-7, is carcinogenic to rat lungs when inhaled over the course of 2 years, but does not induce pleural mesothelioma. In contrast, intratracheal instillation of MWCNT-7 causes both lung tumors and pleural mesotheliomas. To explore the possibility of the use of the intratracheal instillation technique in risk characterization, we administered this widely used reference material using instillation to male F344 rats at doses of 0.0175, 0.07, 0.28, or 0.42 mg/kg once per week for 13 weeks, followed by an observation period of 91 weeks and compared the dose–response toxicological data with the 2-year inhalation study. Lung tumors and pleural mesotheliomas were induced in a dose-dependent manner (the 0.42 mg/kg group was excluded from analysis of lung tumor formation). The lung burden of MWCNT-7 at week 13 was dose-dependent: 0.0302, 0.191, 0.98, and 1.369 mg/lung in the 0.0175, 0.07, 0.28, and 0.42 mg/kg groups, respectively. The number of fibers in the pleural lavage fluid was also dose-dependent. The no-observed-adverse-effect-level (NOAEL) of MWCNT-7’s carcinogenicity was 0.0302 mg/lung based on no significant lung tumor induction and no mesothelioma induction in the 0.0175 mg/kg group. When compared based on lung burden, the dose–response relationship and NOAEL of lung tumor induction resembled those in the 2-year inhalation study, offering the possibility of the application of this protocol for risk characterization.

## 1. Background

Carbon nanotubes (CNTs) are cylindrical nanomaterials composed of concentric graphene cylinders. Their exceptional mechanical, electrical, and thermal properties have led to their widespread use in various industrial and biomedical applications. However, increasing occupational and environmental exposure has raised concerns regarding their potential adverse health effects, particularly to the respiratory system [1,2,3,4]. Over the past decade, a mounting number of studies have documented CNT-induced pulmonary toxicities (inflammation, fibrosis, and granuloma formation), carcinogenicity (lung cancer and mesothelioma), and genotoxicity (reviewed in [5,6,7,8]). Pulmonary toxicity of nanomaterials has been explored mainly by rodent inhalation experiments based on OECD test guidelines such as TG 412, 413, and 451. Besides inhalation, the intratracheal instillation (ITI) technique, in which rats or mice were administered test articles directly through the trachea under deep anesthesia, has been widely employed for hazard identification and mechanistic studies, which have accelerated the development of nanotoxicology since the 2000s. Regarding the carcinogenicity of CNTs, early carcinogenicity studies revealed that a rod-like shaped multiwalled carbon nanotube (MWCNT), MWCNT-7, induced peritoneal mesothelioma in rodent intraperitoneal (i.p.) injection and intrascrotal injection models [9,10,11]. These studies, along with a 15-day inhalation study using the initiator methylcholanthrene that reported the cancer-promoting potential of inhaled MWCNT-7 [12], led the International Agency for Research on Cancer to classify this material in 2014 as belonging to Group 2B (carcinogenic in experimental animals and possibly carcinogenic to humans) [13]: the classification of MWCNT-7 as a group 2B material was published in IARC Monograph Volume 111 in 2017. In 2016, after the classification of MWCNT-7 as a group 2B material, Kasai et al. reported the results of a 2-year whole-body inhalation study in rats which established that MWCNT-7 was a pulmonary carcinogen in rats [14].

A series of i.p. injection studies demonstrated that the mesothelioma-inducing potential of CNTs was closely related to their shapes: straight, thick fibers with sufficient diameter to maintain a rigid rod-like shape-induced peritoneal mesothelioma, whereas thin, flexible fibers did not, consistent with Pott’s fiber paradigm [11,15,16,17,18]. Based on these studies, high-aspect-ratio MWCNTs have been recognized as high-risk materials, and the revised EU CLP Regulation 2024/2564 mandated the labeling of high-aspect-ratio CNTs (with diameters ranging from 30 to 3000 nm and lengths exceeding 5 μm) as Carcinogenic Category 1B materials in 2024.

However, the regulatory framework for CNTs based on their aspect ratio may not be adequate [19]. A recent rat i.p. injection study with large sample sizes suggested that some thin and/or short MWCNTs have weak but non-negligible mesotheliomagenicity [20]. In addition, a series of 2-year carcinogenicity studies using ITI methods in rats demonstrated that thick, rod-like shaped fibers with a high aspect ratio induced lung tumors and/or mesothelioma (MWCNT-7 and MWCNT-N) [21,22,23], whereas much thicker fibers did not significantly induce either type of tumor in rats (MWCNT-A and VGCF-H) [24,25], despite their high aspect ratio. Regarding lung carcinogenicity, it is noted that instillation of very thin (<30 nm), tangled CNTs, often described as having a “cotton candy-like” shape, resulted in significant increases in lung tumors in rats (single-walled carbon nanotubes [SWCNT], double-walled carbon nanotubes [DWCNT], and MWCNT-B) [24,26,27] [Naiki-Ito et al., under review; Particle Fibre Toxicology]. Initial sub-chronic ITI studies demonstrated that the inflammatory response to CNTs was well correlated with BET surface area; thus, tangled agglomerates of SWCNTs and DWCNTs were more toxic than some rod-like shaped MWCNTs [28]. A meta-analysis of genotoxicity studies also revealed that the genotoxic potential of SWCNTs (CNI and Sigma 636797, which are very thin and flexible and form tangled agglomerates) was comparable to that of MWCNT-7 [29]. Besides the shape, CNTs exhibit diverse physicochemical characteristics, including surface reactivity, residual heavy metal content, and surface functionalization. Although the mechanisms by which these parameters contribute to carcinogenesis remain unclear, the carcinogenic potential of CNTs is thought to be associated with chronic inflammation and reactive oxygen species production, similar to asbestos [30,31,32,33,34,35]. Some studies have proposed that the genotoxicity of MWCNTs may be indirect, mediated by inflammation-induced oxidative stress rather than direct interaction with DNA [36,37,38,39].

Although the carcinogenic mechanisms of CNTs appear to be associated with chronic inflammation, adverse outcome pathways (AOPs) specific to CNT-induced carcinogenicity have rarely been proposed [40,41,42], and appropriate endpoints remain undefined. Thus, neither efficient medium-term carcinogenicity tests nor in vitro assays applicable to the risk assessment of CNT-related chronic toxicity have been established. Toxicologists in industry or regulatory agencies, thus, are required to conduct 2-year inhalation studies to evaluate the carcinogenicity of these nanomaterials. However, this is not considered a realistic option for numerous newly developed materials, as the development of inhalation exposure systems is technically complex and resource-intensive, and requires specialized equipment and facilities. As for CNTs, to date, only one 2-year inhalation study has been reported [14], and there is a notable lack of literature even on 90-day inhalation studies [43,44,45]. In this context, we have investigated whether the ITI technique is applicable for the risk assessment of nanomaterials as an alternative to the 2-year inhalation test. Several comparative studies have demonstrated that ITI can capture toxicological effects similar to inhalation in histopathological, transcriptomic, and genotoxic analyses [46,47,48,49]. In addition, a 2-year experimental protocol featuring an ITI technique has identified the carcinogenic hazards of various CNTs (reviewed in [8,50] and referred to in the previous paragraph). Despite the accumulation of a wide range of toxicological data, the ITI and other bolus exposure techniques (e.g., pharyngeal aspiration or intranasal instillation) are generally considered inappropriate for risk assessment, as they involve higher dose rates than inhalation exposure [51] and lack airborne concentration data. However, we hypothesized that the results of 2-year ITI studies could be useful for the risk characterization, if appropriate information is obtained—specifically, the dose–response of carcinogenicity in both ITI and inhalation experiments with a comparable metric.

Recently, we reported an intermittent ITI study in which MWCNT-7 was administered every 4 weeks over 2 years (a total of 26 administrations), aiming to mitigate bolus effects and replicate the time-dependent changes in the lung burden observed in the inhalation study by Kasai et al. [14,23]. This intermittent ITI protocol demonstrated for the first time that intratracheally instilled MWCNT-7 could induce rat lung tumors comparable to those observed in an inhalation study. However, it was insufficient to clarify a dose–response relationship for lung tumor development because only two dose groups were included and the total administered dose was substantially higher than that used in the inhalation study [23].

By taking into account the fact that the carcinogenic effects of inhaled CNTs are driven by CNT fibers that persist in the pulmonary tissue, we propose that it is possible to compare the carcinogenicity data from two experiments with different exposure methods (inhalation and ITI) based on lung burdens (defined here as the amount of test material retained in the lung). Thus, the present study aimed to clarify dose–response relationships for the induction of lung tumors and pleural mesothelioma following MWCNT-7 exposure using a repetitive ITI protocol. We administered three different doses of MWCNT-7 to rats, with lung burden expected to reach a level comparable to that in the inhalation study by Kasai et al. [14]. A fourth higher total dose was also administered to rats in an additional group. This extra-high dose was equivalent to a dose group reported in a previous 2-year ITI study by Numano et al., in which almost all rats developed pleural mesothelioma [22]. In the present study, repetitive ITI was performed once a week for 13 consecutive weeks at the beginning of the 2-year experimental period, rather than using a 2-year intermittent instillation protocol as in our previous study [23]. The administration protocol used in this study resulted in peak MWCNT-7 lung burdens (at week 13) which were roughly consistent with the peak MWCNT-7 lung burdens reported in the studies by Kasai et al. [14] and Numano et al. [22]. Notably, rats exposed to MWCNT-7 developed both lung tumors and pleural mesotheliomas in a dose-dependent manner.

## 2. Materials and Methods

### 2.1. Preparation of the Test Material

The MWCNT, MWCNT-7 (also known as MWNT-7 and Mitsui-7) was obtained from Hodogaya Chemical (Tokyo, Japan). Because the ITI technique bypasses the upper airway, it may allow fiber aggregates, otherwise retained in the nasal passages during inhalation exposure, to reach the lung parenchyma directly. To prevent aggregate formation, the bulk materials were preprocessed using a filtration method, the Taquann method, which removes aggregates and agglomerates without altering the fiber size distribution. This method involves fine filtration through a 53 µm mesh and critical point drying, as described by Taquahashi et al. [23,52]. The Taquann-treated MWCNT was subsequently heated at 200 °C for 2 h in a dry heat sterilizer to eliminate endotoxin contamination. The MWCNT was then dispersed in saline containing 0.1% Tween 80, followed by sonication for 30 min in an ultrasonic bath (100 W; US-702, SND, Suwa, Japan). To avoid precipitation or aggregation of the suspension, the sample was sonicated again in the animal facility immediately prior to administration.

### 2.2. Characterization of the Test Material

The suspension for administration was diluted by purified water containing 0.1% Triton X-100 at a concentration of 1 μg/mL. A 1 µL aliquot of this suspension was applied to an inorganic aluminum oxide membrane filter (Whatman^®^ Anodisc, Cytiva, Marlborough, MA, USA). After gold coating, the filter was examined using a scanning electron microscope (SEM; Quanta™ FEG250, Thermo Fisher Scientific, Waltham, MA, USA) operated at an accelerating voltage of 10–20 kV. A total of 1000 MWCNT fibers were randomly imaged at a magnification of ×5000 for length measurements, and 500 fibers were imaged at ×60,000 for width measurements. Morphological classification and dimensional analysis were conducted using ImageJ software, version 1.54 (National Institutes of Health, Bethesda, MD, USA).

The hydrodynamic diameters of the suspension containing MWCNT at 0.01 mg/mL was measured using dynamic light scattering (DLS; Zetasizer Nano, Malvern, Worcestershire, UK). The suspension was dispensed into 10 mm cuvettes and measured for 70 s. The particle size distribution of the scattering intensity was obtained from three replicate measurements.

The elemental composition of MWCNT was analyzed using an ICP-MS (ICPMS-2030, Shimadzu, Kyoto, Japan).

### 2.3. Animals and Treatment

Male Fischer 344 rats (F344/DuCrlCrlj) were chosen for this study because of their susceptibility to the development of lung tumors [14] and mesotheliomas [25], and to be able to compare the present study with previous ITI studies [21,22,26,27]. A total of 220 five-week-old, specific pathogen-free rats were purchased from Jackson Laboratory Japan (Yokohama, Japan). The rats were housed in polycarbonate cages (2–3 rats per cage) in a room maintained at 23.0 ± 0.3 °C and 49.7 ± 6.5% relative humidity, under a 12-h light/dark cycle with a ventilation rate of more than 10 air changes per hour. Animals were provided with a standard CE-2 basal diet (CLEA Japan, Tokyo, Japan) and filtered drinking water ad libitum. The animal experiment followed the Animal Research Reporting of In Vivo Experiments guidelines [53] and was approved by the Animal Experiment Committee of the Tokyo Metropolitan Institute of Public Health.

Animals were randomly divided into 5 groups using body weight-stratified randomization: vehicle control group (0 mg/kg), low-dose group (0.0175 mg/kg), middle-dose group (0.07 mg/kg), high-dose group (0.28 mg/kg), and extra-high-dose group (0.42 mg/kg), which consisted of 40, 40, 40, 40, and 20 rats, respectively. The extra-high-dose group was included as an additional group outside the main dose ratio (4:1) groups in order to replicate the findings of a previous study by Numano et al., in which 95% of rats (18 out of 19) developed pleural mesothelioma and died [22]. Thus, the number of animals in the extra-high-dose group was reduced to 20 in consideration of the 3Rs principle. Satellite animals (n = 10 per MWCNT-treated group) were additionally included to measure lung and pleural burdens of the MWCNT. The MWCNT dosage was determined based on findings from previous experiments: the lung burden of MWCNT-7 typically decreases by approximately half within one day after a single administration across a wide range of doses (unpublished data; also see [54,55]). However, repeated administration of higher doses gradually impairs the lung’s clearance capacity [23].

Rats were dosed once a week for a total of 13 weeks, starting at 10 weeks of age, via ITI as previously described [23,50]. Briefly, rats were anesthetized with 3.0% isoflurane (Pfizer, NY, USA) and placed on a holder at a 45° incline. A light was shone on the throat, and the larynx was visually inspected. An aerosolizer (MicroSprayer^®^, IA-1B, Penn-Century, which is no longer available; however, a similar type of aerosolizer is supplied by Natsume Seisakusho, Tokyo, Japan) connected to a 1 mL syringe (HJ5010-LL, Osaka Chemical, Osaka, Japan) was inserted into the trachea, and the vehicle or MWCNT suspension was delivered into the lungs by pressing the syringe plunger. The administration volume was set at 1 mL/kg body weight, adjusted for each animal based on its body weight measured on the morning of the day of administration. To minimize a potential confounder due to the order of instillation, we changed the order of the groups treated each instillation day.

General health status was monitored twice daily, and body weight was recorded weekly. After the 13-week instillation period, all animals were maintained for an additional 91 weeks for carcinogenicity assessment (104-week-long experimental period). Of the 10 satellite animals assigned to each MWCNT-treated group, 5 rats were sacrificed one day after the 13th instillation to measure peak lung burden and the number of fibers in the pleural cavity, while the remaining 5 rats were sacrificed at week 104. The disposition of the animals is shown in Table S1.

### 2.4. Autopsy and Sample Collection

At the end of the study (week 104), surviving animals were euthanized by exsanguination via the abdominal aorta under 3.0% isoflurane anesthesia, followed by gross pathological examination. An aliquot of the collected blood was used for hematological analysis, and the remaining portion was processed immediately to obtain serum.

Bronchoalveolar lavage fluid (BALF) was collected from 8 animals per group (Table S1). To selectively lavage the right lung, the left bronchus was ligated using surgical thread, and the right lung was lavaged twice with 4 mL of phosphate-buffered saline (PBS; pH7.2) at a water pressure of 30 cm. The collected lavage fluid was centrifuged at 402× *g* for 10 min at 4 °C. The supernatant was used for biochemical assays, and the cell pellet was gently resuspended for cytological analysis.

For quantification of lung burdens, whole lungs were collected from 5 satellite animals at week 13 and from the remaining satellite animals at the terminal necropsy (Table S1). Pleural lavage fluid (PLF) was obtained from the same animals used for lung burden measurement at both necropsy time points. The thoracic cavity was lavaged once with 8 mL of saline using a syringe fitted with a 22-gauge needle, and the retrieved fluid was processed for SEM analysis.

At the terminal necropsy, the brain, lungs, liver, spleen, kidneys, and adrenal glands were excised from 8 animals per group and weighed (Table S1). Heart weights were obtained from satellite animals, as the hearts of the main animals were not removed in order to ensure accurate BALF collection and optimal lung fixation for histopathological analysis. Major organs including the trachea, lungs, parietal pleura (diaphragm and chest wall), heart, spleen, mediastinal lymph nodes, thymus, tongue, salivary glands, esophagus, stomach, small and large intestines, liver, pancreas, kidneys, urinary bladder, adrenal glands, pituitary gland, thyroid and parathyroid glands, testes, epididymides, seminal vesicles, prostate, preputial glands, mammary glands, muscle (quadriceps femoris), femur, brain, spinal cord, eyes, Harderian glands, Zymbal’s glands, and skin were harvested from all animals for histopathological evaluation. The data for organs with abnormal sizes, such as the spleens and livers affected by lymphoma, or the kidneys and adrenal glands harboring tumors, were excluded from the statistical analysis of organ weights.

Animals that died or were euthanized for humane reasons prior to terminal necropsy were similarly treated except collecting BALF and PLF was not performed.

### 2.5. Hematological Analysis

At terminal necropsy, blood samples were collected from 8 animals in each group (Table S1) and analyzed using an automated hematology analyzer (pocH-100iv, Sysmex, Kobe, Japan). Differential leukocyte counts were microscopically determined from smears stained by the standard May–Grunwald–Giemsa method.

### 2.6. Histopathology

All dissected organs and tumor masses were fixed in 10% neutral-buffered formalin, embedded in paraffin, sectioned at a thickness of 4 μm, and stained with hematoxylin and eosin. All 5 lung lobes were examined individually with a total of 9 sections: 4 transverse sections of the left lobe, 1 transverse section of the right cranial lobe, 1 longitudinal horizontal section of the right middle lobe, 2 transverse sections of the right caudal lobe, and 1 transverse section of the accessory lobe. Regarding mesothelial tissues, 3 transverse sections of the ventral chest wall, 3 transverse sections of the dorsal chest wall, 3 sections (dorsal–ventral axis) of the diaphragm, and 1 longitudinal section of the mediastinum with the heart were examined.

The severity of non-neoplastic lesions was assessed using a four-tier grading scale: no/minimal (0), slight (1), moderate (2), and marked (3) based on the observation of all examined sections. Proliferative lesions in the lung and pleura were blindly reviewed by 3 pathologists, and the final diagnosis was made by Dai Nakae, one of the authors, who is a board-certified pathologist by the Japanese Society of Toxicologic Pathology, the Japanese Society of Pathology, and the International Academy of Toxicologic Pathology. Pleural mesotheliomas were categorized into 3 histological types: epithelioid, sarcomatoid, and biphasic. Biphasic mesothelioma was diagnosed when epithelioid and sarcomatoid components each comprised at least 10% of the tumor tissue collected from the thoracic cavity [56].

### 2.7. Cytological Analysis for the BALF

Cells obtained from the centrifuged BALF were stained with Turk’s solution, and leukocyte numbers were manually counted immediately using a hemocytometer slide. For differential leukocyte analysis, cytological slides were prepared (CF-120; Sakura Finetek Japan, Tokyo, Japan), stained with May–Grunwald–Giemsa, and examined using the same method as for hematological analysis.

### 2.8. Biochemical Analysis of the BALF

Lactate dehydrogenase (LDH) activity in the BALF was measured using an assay kit from Takara Bio (Shiga, Japan) along with an LDH calibration standard (Roche Diagnostics, Mannheim, Germany). The total protein content in the BALF supernatant was determined using a kit from Cytiva (MA, USA) following the manufacturer’s recommended protocol.

### 2.9. Measurement of Serum Mesothelin Level

Serum N-ERC/mesothelin levels were measured using an ELISA kit (IBL, Gunma, Japan) in 35 rats without pleural mesothelioma at week 104 (8 from the control group, 7 from the low-dose group, 10 from the middle-dose group, 8 from the high-dose group, and 2 from the extra-high-dose group). N-ERC/mesothelin was also measured in the rats with pleural mesothelioma that were necropsied at week 104 (2 from the middle-dose group and 2 from the high-dose group) and from 6 rats in the high-dose group and 9 rats in the extra-high-dose group that were sacrificed during the observation period. The sera obtained from rats diagnosed with peritoneal mesothelioma were not included in the analysis.

### 2.10. Quantification of MWCNT in the Lung and PLF

At weeks 13 and 104, MWCNT amounts in the lungs and PLF from satellite animals were quantified (Table S1). MWCNT quantification in the lungs was performed as previously described [57]. In brief, formalin-fixed whole-lung samples were digested using a strong alkaline solution (Clean 99, K200; Clean Chemical, Osaka, Japan). After organic material was removed with sulfuric acid, benzo[ghi]perylene (B(ghi)P) (B9009, Merck KGaA, Darmstadt, Germany) was added to the solution containing the MWCNTs. The sample was then thoroughly mixed to facilitate B(ghi)P adsorption onto the MWCNTs. The solution was filtered through a membrane (Whatman^®^ Nuclepore, 111109, Cytiva), and the B(ghi)P adsorbed on the MWCNTs was desorbed into acetonitrile. The B(ghi)P in the acetonitrile was analyzed using an ultra-high-performance liquid chromatography (UHPLC) system (Nexera X2, Shimadzu, Kyoto, Japan).

The numbers of MWCNT fibers in the PLFs were counted using SEM, following previously established protocols [14,23]. The PLF samples were centrifuged at 20,000× *g* for 1 h, and the resulting pellet was digested with Clean 99 for 2 h. After washing, the sample was resuspended in 50 µL of purified water containing 0.1% Triton X-100. A total of 400–700 fibers were analyzed for each sample.

### 2.11. Statistical Analysis

Body weights, organ weights, hematological parameters, cell counts in the blood and BALF, BALF biochemistry, and serum mesothelin levels were analyzed using Dunnett’s multiple comparison test. Data normality was assessed by Kolmogorov–Smirnov tests. Survival curves were generated using the Kaplan–Meier method, and statistical differences in survival rates between treated and control groups were assessed by the log-rank test. Cause-specific survival of rats that developed mesothelioma, i.e., the length of time of survival of rats that developed mesothelioma, was also evaluated by the log-rank test. Differences in the incidence of neoplastic and non-neoplastic lesions compared with the control group were evaluated using Fisher’s exact test. For the main 4 groups (control, low-dose, middle-dose, and high-dose groups), the incidences of lung tumors and mesotheliomas were also analyzed for their dose-related trends using a survival-adjusted Poly-K test (K = 3). Histological scores of non-neoplastic lesions were analyzed using Steel’s multiple comparison test. Differences in fiber length and diameter between bulk MWCNT and Taquann-treated MWCNT, lung burden, and number of MWCNT fibers in the PLF were analyzed using Student’s *t*-test. Serum mesothelin levels were assessed using the Tukey–Kramer test and Jonckheere’s trend test.

Statistical significance was based on *p*-values below the threshold of 0.05. All statistical analyses were conducted as two-tailed tests except the Poly-K test for carcinogenicity. Statistical analyses were performed using StatLight software, version 2.0 (Yukms, Kawasaki, Japan), EXCEL Statistics, version 7 (Esumi, Tokyo, Japan), or R software with the ToxicR package, version 1.1.2).

## 3. Results

### 3.1. Characterization of the MWCNT

**Figure 1:**
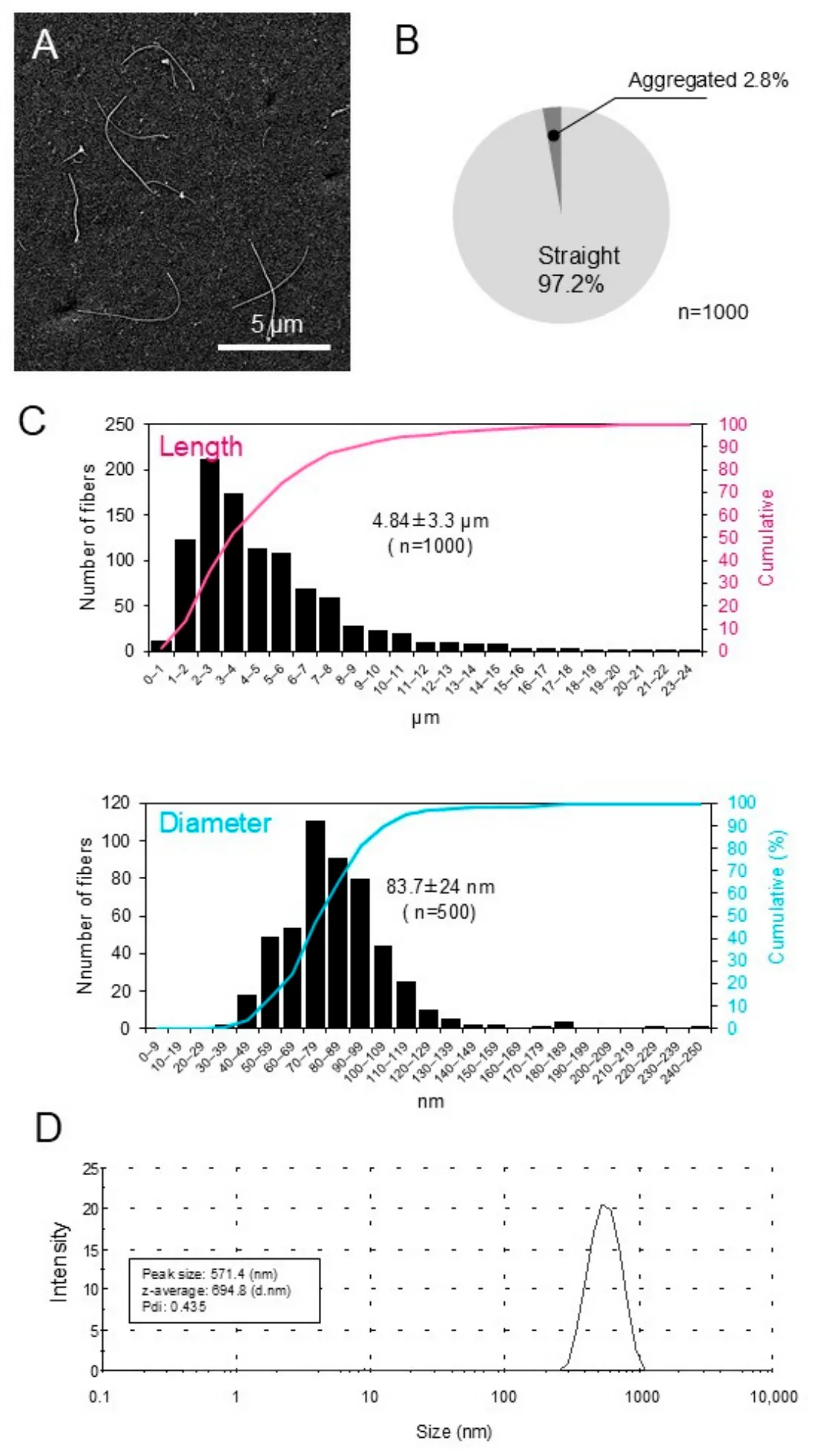
Characterization of the test material. (**A**) SEM image of Taquann-MWCNT-7. (**B**) Morphology of individual fibers. Singular, straight-type fibers represent 97.2% of the total number of particles examined. (**C**) Distribution of fiber length and diameter of Taquann-MWCNT-7. (**D**) DLS analysis. The peak hydrodynamic diameter is 571.4 nm (z-average: 694.8 d.nm.; polydispersity index: 0.435).

A representative SEM image of Taquann-MWCNT-7 fibers suspended in the administration medium is shown in Figure 1A. Most fibers exhibited a singular, straight structure (97.2%), with only a small percentage displaying aggregation (2.8%) (Figure 1B). The average length and diameter of the MWCNT-7 fibers was 4.8 µm and 83.7 nm (Figure 1C) respectively, which were not different from the bulk MWCNT-7 (Figure S1). DLS analysis revealed that only a single peak of the particle size distribution was observed in the MWCNT suspension. The z-average size of Taquann-MWCNT-7 in suspension and the polydispersity index was 694.8 d.nm and 0.435, respectively (Figure 1D). This indicates that the particle size distribution has a relatively wide range. ICP-MS analysis revealed that MWCNT-7 had low levels of metal impurities such as iron (Fe) (Table S2).

### 3.2. Body Weights, Survival Rates, Organ Weights, and Hematological Analysis

**Figure 2:**
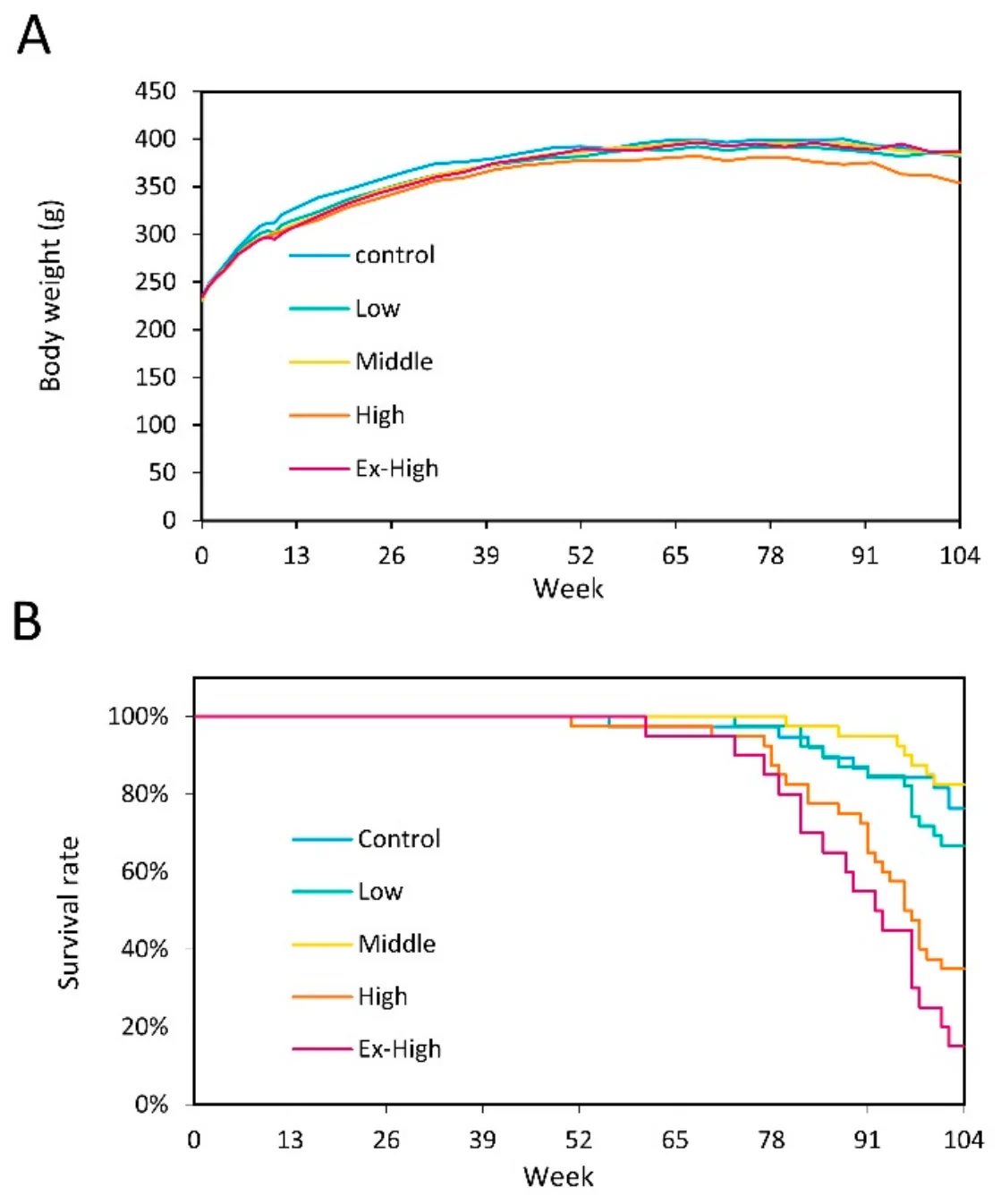
Growth and survival rates. (**A**) Body weight curves. Slight growth retardation occurred in the MWCNT-7-treated groups during the administration period and a significant suppression of weight gain was observed in the high-dose group throughout the experimental period. (**B**) Kaplan–Meier survival plot. Survival rates started decreasing around week 75 in the high-dose and the extra-high-dose groups due to death from pleural mesothelioma. Significant differences were detected between the both of these MWCNT-7-treated groups and the control group.

Body weight curves are presented in Figure 2A. During the administration period, slight growth retardation was observed in the MWCNT-7-treated groups, compared with the control group. The body weights of all treated groups were significantly lower than that of the control group at week 13, but the suppression levels in body weight gain were only 2.8%, 4.1%, 6.0%, and 6.5% in the low-dose, middle-dose, high-dose, and extra-high-dose groups, respectively, which were well below the threshold for a maximum tolerated dose (10% suppression). The differences gradually decreased over the observation period. At week 104, body weight was significantly lower only in the high-dose group compared to the control group (Table 1).

Three rats died from choking on pelleted food resulting in severe respiratory distress and were excluded from the study (two rats in the control group and one rat in the low-dose group; Table S1). All of the remaining rats were included in the study. Six rats were found dead during the study, three in the low-dose group and one each in the middle-dose, high-dose, and extra-high-dose groups (Table S3). The health status of the rats was monitored twice daily and these rats were found shortly after death and were included in the pathological analysis. Animals that died or were euthanized before week 104 are listed in Table S3. Two satellite animals died or were euthanized before the end of the observation period (one died from choking on food, and the other bearing lymphoma); thus, lung burden and fiber counts in the PLF were analyzed with n = 4 in the middle-dose and extra-high-dose groups (see Table S1).

Survival curves are shown in Figure 2B. Significant decreases in the average survival time were observed in the high-dose group and the extra-high-dose group, compared with the control group. The first rat that developed pleural mesothelioma was observed in the extra-high-dose group at week 73 (Table S3). Pleural mesothelioma caused rapid body weight loss, anemia, and labored breathing in affected rats. At autopsy, most rats with pleural mesothelioma exhibited cardiac tamponade and/or hemorrhage in the pleural cavity; these symptoms could be predicted while the animals were alive by palpating the chest (i.e., difficulty in detecting heartbeats). Survival rates in the high-dose and extra-high-dose groups gradually declined due to mesothelioma-related deaths. At terminal necropsy, survival rates in these two groups were significantly lower than in the control group (Figure 2B and Table 1). It was also found that the cause-specific survival of rats that developed pleural mesothelioma was lower in the extra-high-dose group (average survival: 92.0 weeks) than in the high-dose group (average survival: 96.2 weeks) but without statistical significance.

Table 1 shows lung weights at week 104. Absolute lung weight was significantly increased in the high-dose group. In the extra-high-dose group, lung weight was increased and the increase was deemed to be biologically significant; however, the increase in lung weight in this group was not statistically significant. Relative lung weight was significantly increased in both the high-dose and extra-high-dose groups. No treatment-related changes in organ weights were observed in other organs (Table S4).

No significant differences in hematological parameters were found between the MWCNT-7-treated groups and the control group (Table S5).

### 3.3. Macroscopic Findings

**Figure 3:**
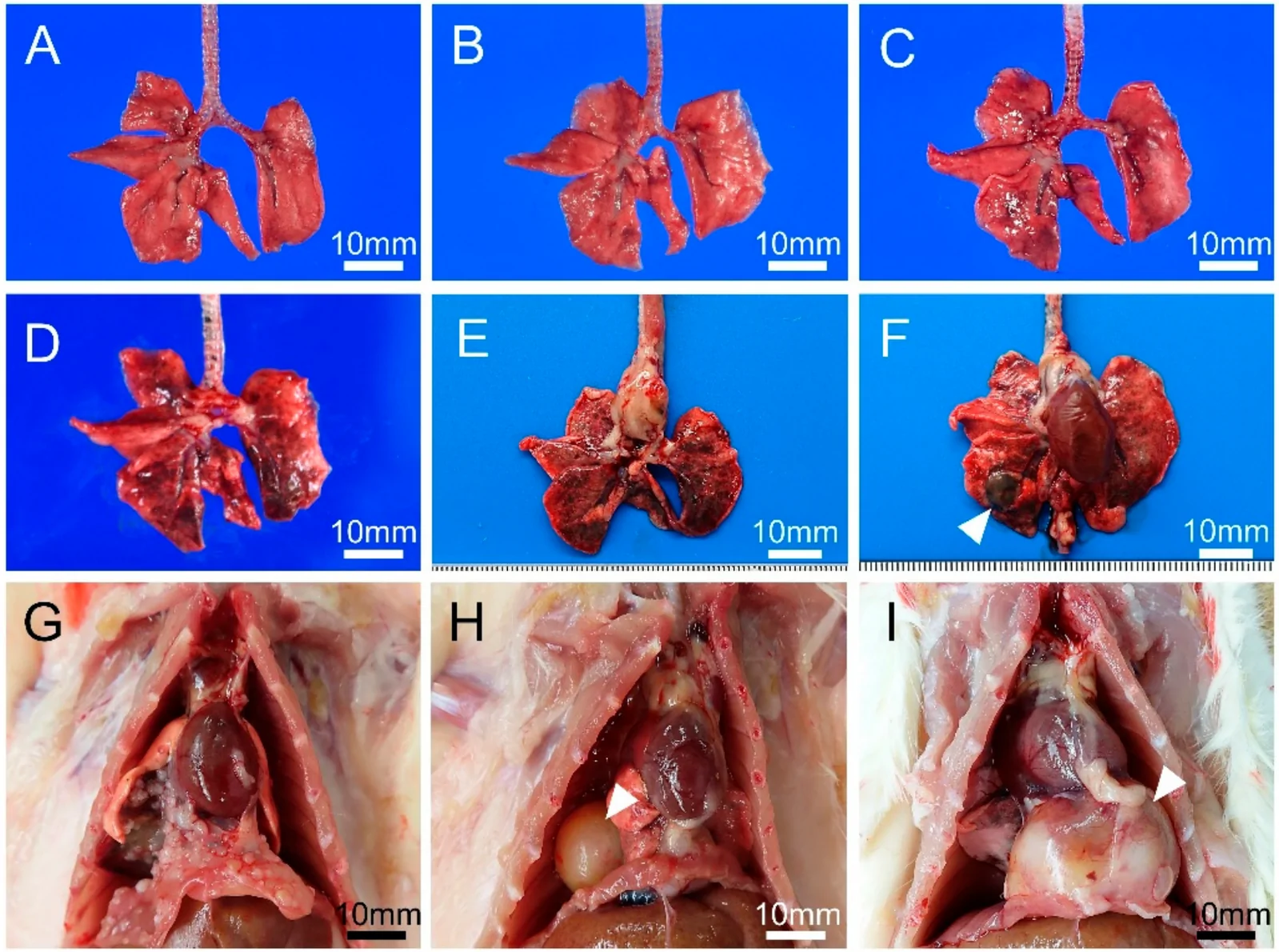
Gross appearance of lungs at the terminal sacrifice and of pleural mesotheliomas in MWCNT-7-treated rats. (**A**–**E**) Representative images of rat lungs in the control (**A**), low-dose (**B**), middle-dose (**C**), high-dose (**D**), and extra-high-dose (**E**) groups. Surfaces of the lung parenchyma appear gray to black, with the darker coloration occurring in a dose-dependent manner. More pronounced blackening is observed near the hilum of each lobe, in the entire accessory lobe, and in the posterior side of the left lobe and the right posterior lobe. (**F**) A large, gray nodule on the right lobe in a rat in the extra-high-dose group sacrificed at week 104 (arrowhead). (**G**–**I**) Mesotheliomas found in MWCNT-treated rats. Numerous small nodules widely distributed on the parietal pleura (mainly on the diaphragm and mediastinum) and the visceral pleura in a high-dose rat sacrificed at week 91 (**G**). A large, yellowish-white nodule on the surface of the diaphragm (arrowhead) in an extra-high-dose rat sacrificed at week 92 (**H**). A large white nodule (arrowhead) formed in the posterior mediastinal area (within the RPFs) in an extra-high-dose rat sacrificed at week 77 (**I**).

The lung surfaces of the rats in the middle-dose, high-dose, and extra-high-dose groups appeared gray to black due to MWCNT fiber deposition. The black tone was dose-dependently darker, and prominent near the pulmonary hilum, in the hilar regions, and on the posterior side of the lobes (Figure 3A–E). Gray-yellowish or dark reddish nodules were also observed on the lung surface of some rats (Figure 3F). Location and size of all lung tumor nodules are summarized in Table S6. One rat in the middle-dose group had two lung tumors.

Macroscopic diaphragmatic lesions were frequently observed in the MWCNT-7-treated groups, e.g., whitish thickening of the central tendon of the diaphragm. The gross appearance of the nodules of pleural mesothelioma varied from numerous small nodules disseminated across the surface of the serosal membrane to a few large nodules, and these nodules were mostly located in the posterior mediastinum, including the retrocardiac pleural folds (RPFs: also see Figure 3 in [23] and diaphragm (Figure 3G–I). Hemothorax and cardiac tamponade were often observed. Some neoplasms penetrated the abdominal cavity and adhered to the liver surface. Gross findings of all mesotheliomas are summarized in Table S7.

One peritoneal mesothelioma was found in the extra-high-dose group (Table S8). This tumor appeared to originate from the diaphragm: small white nodules were spread densely on the surface of the diaphragm, liver and omentum.

In all MWCNT-treated rats, some black spots were observed in the trachea where the tip of the aerosolizer was positioned during administration (e.g., Figure 3D).

### 3.4. Microscopic Findings

**Figure 4:**
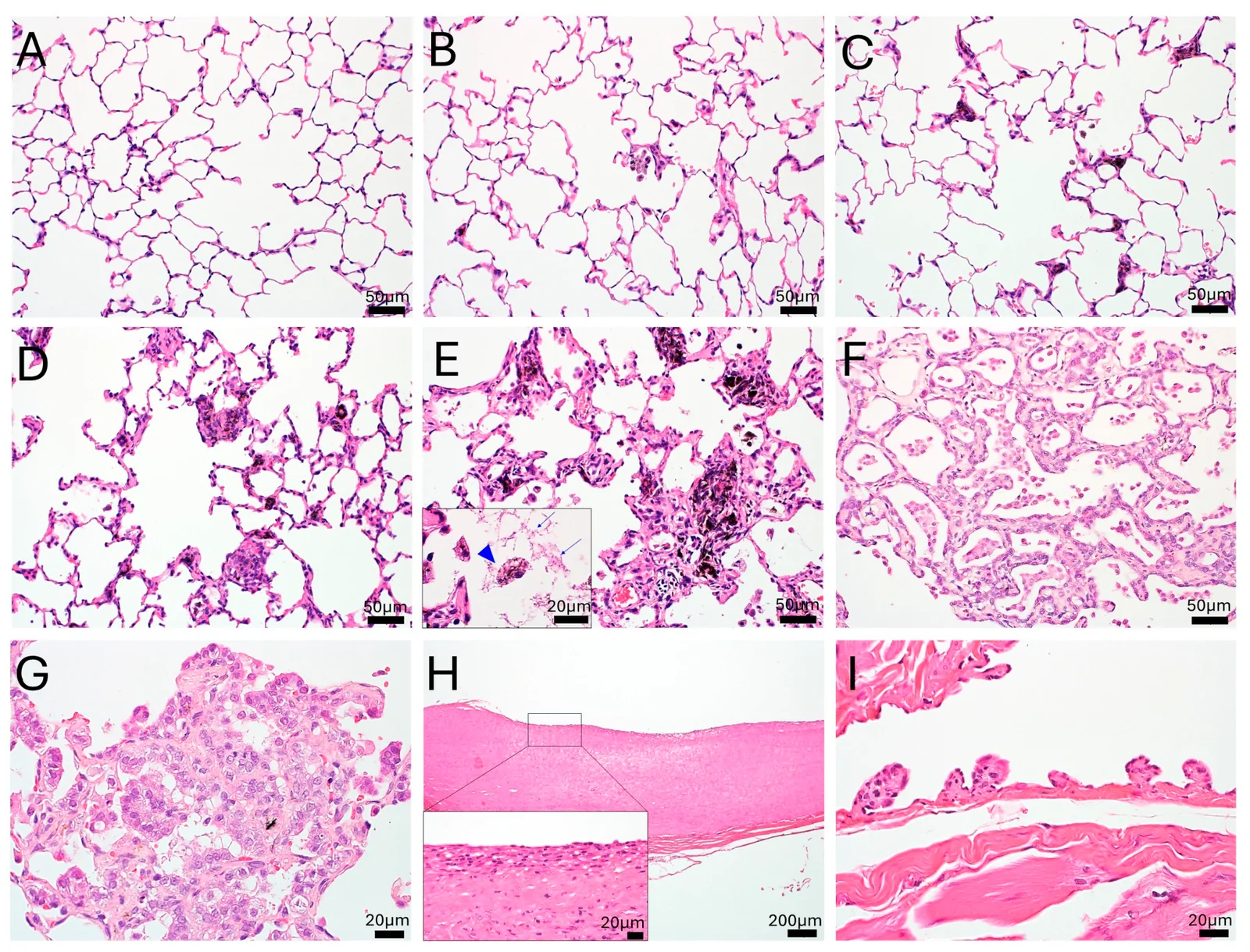
Microscopic findings of non-neoplastic lesions in the lung and pleura of MWCNT-7-treated rats. (**A**–**E**) Representative images of rat lungs in the control (**A**), low-dose (**B**), middle-dose (**C**), high-dose (**D**), and extra-high-dose (**E**) groups. Granulomatous lesions and MWCNT-engulfing macrophages aggregated around alveolar ducts were observed in the high-dose and extra-high-dose groups. Fractured macrophages (inset, arrowhead) and debris (inset, arrow) were frequently found in the alveolar spaces in extra-high-dose group rats. (**F**) Bronchiolo-alveolar hyperplasia (middle-dose group). Single layered epithelial cells have uniformly proliferated along the alveolar walls. Neutrophils and eosinophils are focally accumulated in these alveolar spaces. (**G**) Atypical hyperplasia (high-dose group). Cuboidal epithelial cells have proliferated and formed an irregular structure with some interstitial tissue. (**H**) Focal fibrosis of the diaphragm (middle-dose group). The inset shows thickened collagenous fibers accompanied with fibroblasts, fibrocytes, and leukocytes just beneath a thin, single layer of mesothelial cells covering the surface of this region of the diaphragm. (**I**) Mesothelial hyperplasia (middle-dose group). Mesothelial cells protruding from the surface of the diaphragm showing a papillary pattern.

The results of the histopathological examinations of non-neoplastic lesions in the lung and pleura are presented in Table 2 and Figure 4. In the MWCNT-7-treated groups, frequent accumulation of MWCNT-engulfing macrophages and granulomas containing MWCNT fibers were observed in the bronchiolar and alveolar walls, particularly from the regions around the terminal bronchioles to the alveolar ducts (Figure 4A–E). Similar macrophage accumulation was also noted in the distal acinus regions including the subpleural zones. The severity of these lesions increased in a dose-dependent manner (Table 2). Fractured macrophages and their debris, including MWCNT fibers, were prominent in the extra-high-dose group (Figure 4E, inset). No marked infiltration of inflammatory cells other than macrophages was observed in any rats in the treated groups.

MWCNT fibers were also observed outside the pulmonary parenchyma without any accompanying inflammatory cells. These fibers were found in the bronchus-associated lymphoid tissue (BALT), mediastinal lymph nodes (as large agglomerates associated with non-inflammatory macrophages), mesothelium of the visceral pleura (as singular or bundle-like fibers penetrating the pleural surface), and subepithelium of the tracheal wall (as components of large granulomas located beneath the mucosal epithelium at the black spots found in the tracheas, consistent with the macroscopic findings).

The presence and severity of fibrosis in the alveolar walls surrounding the alveolar ducts was significantly increased in the middle-dose, high-dose, and extra-high-dose groups (Table 2).

Incidences of bronchiolo-alveolar hyperplasia (Figure 4F) were significantly increased in the middle-dose and high-dose groups (Table 2). The histological distribution of hyperplasia varied depending on the exposure dose. Hyperplasias were found in the proximal and distal acinus regions in the control and low-dose groups, and they were also observed around the terminal bronchioles in the central regions of the lung in the middle-dose and high-dose groups (Table S9 and Figure S2). Atypical hyperplasia, considered a precancerous lesion, was observed in two rats: one in the high-dose group and the other in the extra-high-dose group (Table 2, Figure 4G).

Fibrosis in the mesothelial tissues was prominent throughout the thoracic cavity in both parietal (chest wall and diaphragm) and visceral pleurae, and related serosa (RPFs) of the MWCNT-7-treated rats. Marked fibrotic thickening and infiltration of inflammatory cells were frequently observed in the diaphragm (Figure 4H). In three rats without mesothelioma (two rats in the middle-dose and one rat in the high-dose groups, Table S7), mesothelial hyperplasias were observed as papillary or layered protrusions of mesothelial cells in the diaphragm and chest wall (Figure 4I).

**Figure 5:**
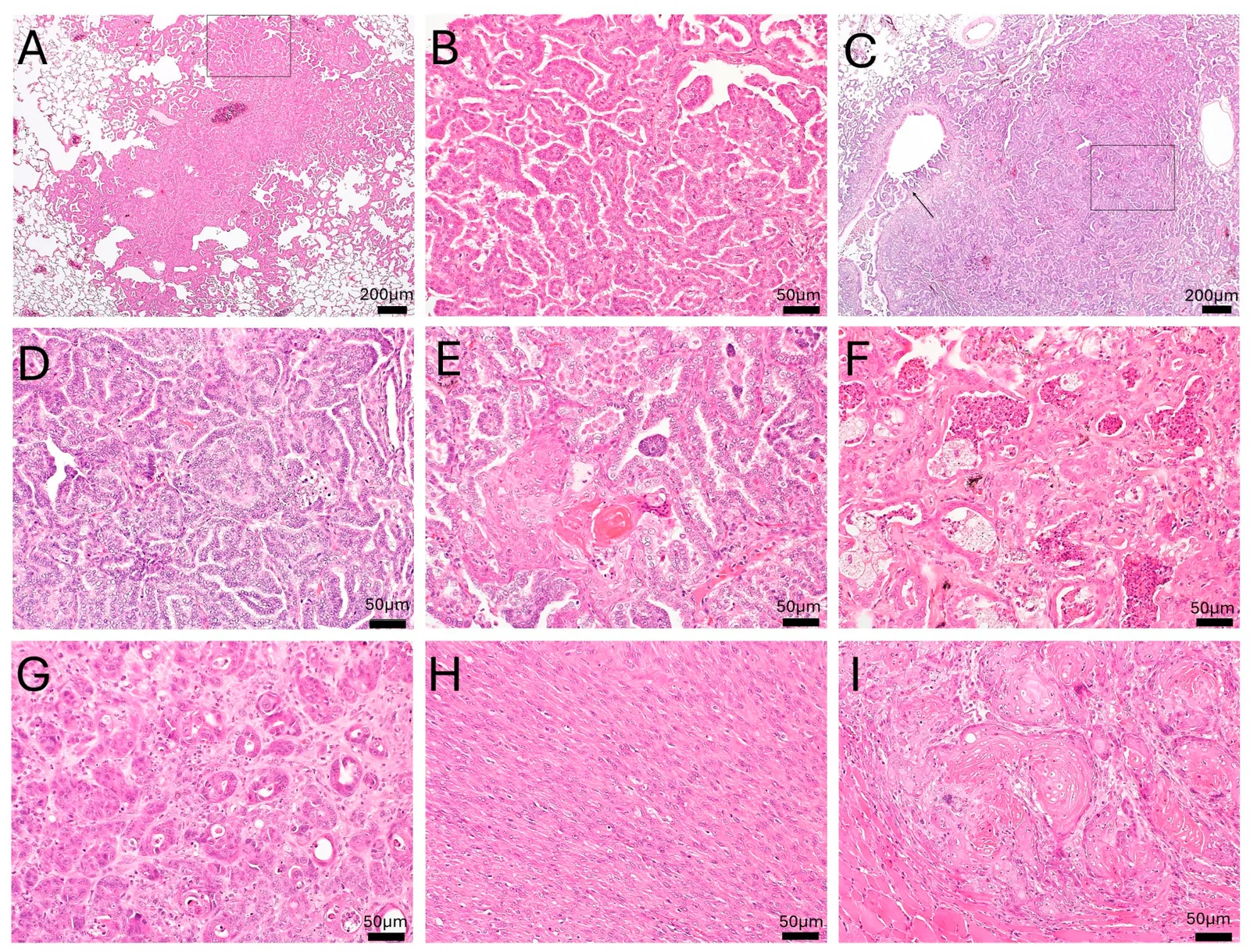
Representative histopathological features of neoplastic lesions in the lungs and pleura. (**A**) Adenoma in a rat in the high-dose group. Epithelial cells have uniformly proliferated without compressing the surrounding alveolar architecture. (**B**) Higher magnification of the insert area in (**A**). Tumor cells have formed a papillary pattern. (**C**) Adenocarcinoma in a rat in the extra-high-dose group. The tumor mass has compressed the surrounding alveolar architecture and some tumor cells have invaded into the bronchus (arrow). (**D**) Higher magnification of the insert area of (**C**). Basophilic tumor cells are irregularly arranged as papillary structures supported by a connective tissue core. (**E**) Adenosquamous carcinoma in a rat in the middle-dose group. Nests of squamous cells containing keratin pearls are intermixed with irregularly arranged cuboidal adenomatous cells. (**F**) Squamous cell carcinoma without overt keratinization in a rat in the high-dose group. Polygonal cells with distinct intercellular bridges are arranged in sheet-like or glandular patterns. Large amounts of neutrophils and cellular debris are contained in the tumor nest. (**G**) Epithelioid mesothelioma is found in the diaphragm of a rat in the extra-high-dose group. Cuboidal epithelial-type tumor cells have formed numerous glandular or acinar-like structures accompanied by numerous interstitial cells. (**H**) Sarcomatoid mesothelioma is found in the diaphragm of a rat in the extra-high-dose group. Spindle-shaped tumor cells with abundant eosinophilic cytoplasm have formed a sheet-like mass. (**I**) Epithelioid mesothelioma with high keratinization in the diaphragm of a rat in the extra-high-dose group. Tumor cells have developed squamous differentiation, formed pearl-like structures, and invaded into muscle fibers of the diaphragm accompanied by inflammatory cells.

The results of the histopathological examination of neoplastic lesions in the lungs and pleura are summarized in Table 3 and Figure 5.

Within a total of 21 animals bearing lung tumors across the groups, 16 cases were found at terminal necropsy, and five cases were found in rats that were necropsied due to other diseases prior to the termination of the observation period (Tables S3 and S6). One rat had a bronchiolo-alveolar adenoma and a bronchiolo-alveolar adenocarcinoma. The incidence of total lung tumors, including adenomas and/or carcinomas, was significantly elevated in the middle-dose (7/40) and high-dose (7/40) groups compared with the control group (1/38) (Table 3). As the survival rate was significantly reduced in the high-dose group due to the development of pleural mesotheliomas (Table 1), the incidence in this group appeared similar to that in the middle-dose group; however, the Poly-3 test revealed a dose-dependent carcinogenic effect on the lung in the main four groups (*p* = 0.0267 for bronchiolo-alveolar adenoma; *p* = 0.0056 for total tumors) (Table 3). Evaluation of lung tumors in the extra-high-dose group was excluded because of the early deaths in this group due to pleural mesothelioma (Table S3). Bronchiolo-alveolar adenomas and adenocarcinomas were observed across all groups, including the control. The adenomas consisted of epithelial cells proliferating in papillary (Figure 5A,B), solid, or mixed patterns. The carcinomas exhibited a round to oval mass, which in some cases were without clear boundaries in the surrounding tissues. Tumor cells exhibited papillomatous and sheet-like proliferation, with frequent mitotic figures and invasion into the surrounding tissues (Figure 5C,D). One rat in the middle-dose group had an adenosquamous carcinoma, characterized by a mixture of adenomatous and squamous components with transitional features between them (Figure 5E). One rat in the high-dose group had a squamous cell carcinoma, consisting of squamous tumor cells without overt keratinization (Figure 5F).

A total of 27 rats in the MWCNT-treated groups developed malignant pleural mesothelioma (Table 3 and Table S7). The incidence of mesothelioma was significantly increased in the high-dose and extra-high-dose groups compared with the control group (Table 3). Most mesotheliomas were advanced and lethal, and thus were found before terminal necropsy. Five rats with mesothelioma survived to the end of the observation period (Table S7). Histologically, all three major types of mesothelioma were detected in this study: epithelioid (seven rats) (Figure 5G), sarcomatoid (17 rats) (Figure 5H), and biphasic (three rats) (Table S7). Intriguingly, mesotheliomas macroscopically recognized as small nodules preferentially exhibited epithelioid features, whereas mesotheliomas recognized as large nodules mainly showed sarcomatoid features. As for an indication of heterologous differentiation of the mesotheliomas, bone formation was observed in four cases of sarcomatoid mesothelioma. Notably, keratinization was observed in two cases of epithelioid mesothelioma (extra-high-dose group; Figure 5I, Table S7). This finding is known as a rare histological feature suggesting an unusual differentiation pattern in human malignant pleural mesothelioma [58] and was never reported previously in CNT-exposed rodents.

The incidences of neoplasms in other organs are summarized in Table S8. The incidence of the peritoneal mesothelioma in each CNT-treatment group was not significantly increased compared with the control. Most peritoneal mesotheliomas were found to have originated from the tunica vaginalis (Table S8). One case of peritoneal mesothelioma in the extra-high-dose group that appeared to originate from the diaphragm, showed an epithelial pattern with cellular and nuclear pleomorphism, and necrosis, without any lesions around the scrotum. In F344 rats, spontaneous peritoneal mesotheliomas with distinctive histological features (e.g., complex papillary structure, plenty stroma, pedunculated fibrovascular stalks) are frequently found [59]. Therefore, the lack of scrotal lesions indicated that this peritoneal mesothelioma was likely to have been induced by MWCNT. None of the other neoplasms were MWCNT-7-treatment-related.

### 3.5. Cytological and Biochemical Analyses of BALF

**Figure 6:**
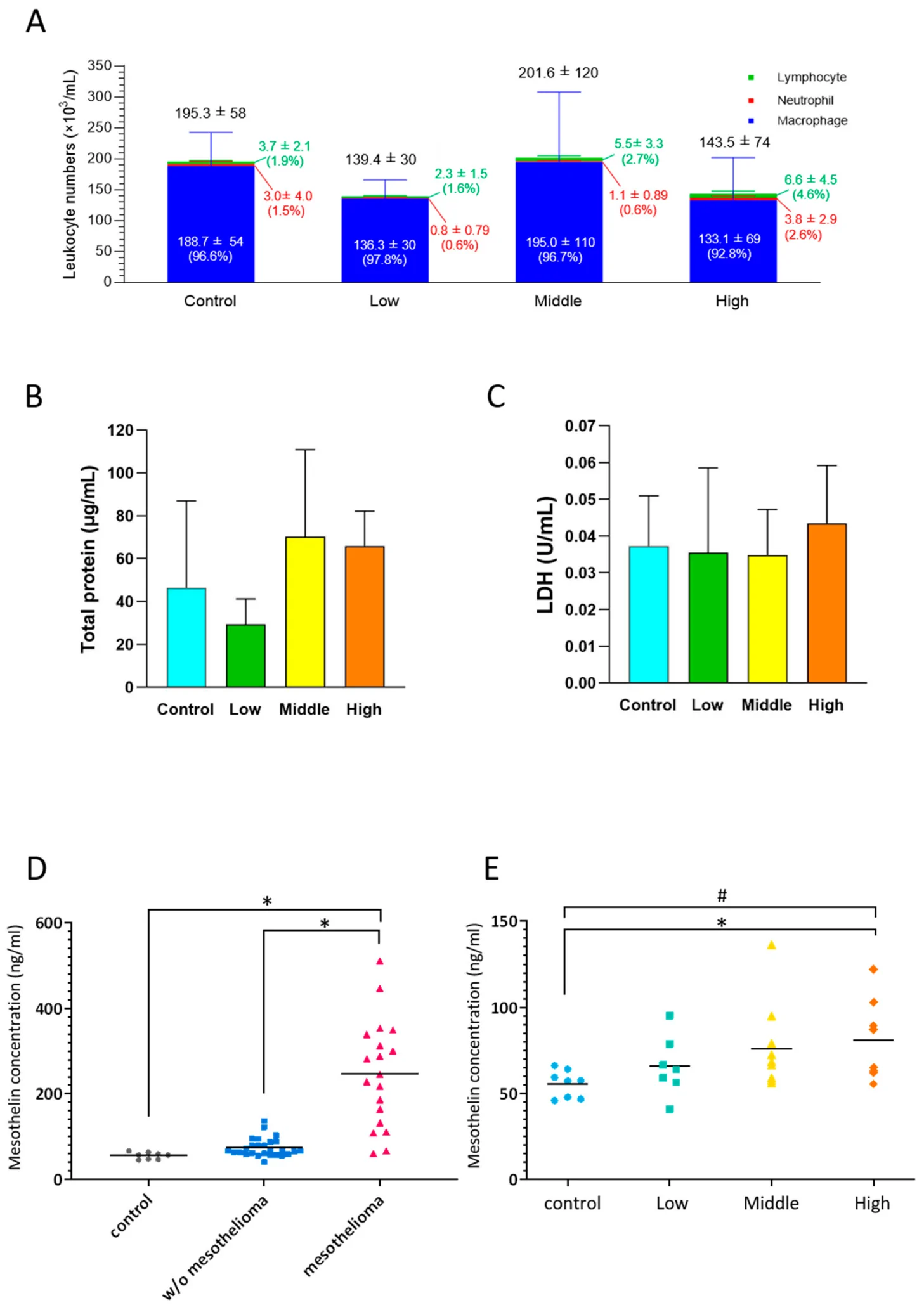
Cytological and biochemical analyses of the BALF and serum mesothelin concentration. (**A**) Differential leukocyte analysis of the BALF at week 104 (n = 8). The number of lymphocytes, neutrophils, and macrophages along with standard deviations are shown in the stacked graph. The total leukocyte number and standard deviations are shown at the top of each graph. Parentheses show the percentages of each cell type. (**B**) Total protein concentration of the BALF at week 104 (n = 8). (**C**) LDH activity in the BALF at week 104 (n = 8). Error bars show standard deviations. Differences in the values between groups were evaluated by Dunnett’s test. (**D**,**E**) Concentration of N-ERC/mesothelin in the serum. Comparison among the control rats (n = 8), the MWCNT-7-treated rats without mesothelioma (n = 27), and MWCNT-7-treated rats with mesothelioma (n = 19) (**D**). The 2 latter data sets in (**D**) include rats from all treated groups. Comparison among rats not bearing mesotheliomas in the control, low-dose, middle-dose, and high-dose groups (**E**). The data from the extra-high-dose-group was excluded because there was only one rat without mesothelioma. Bars show mean values. * Significant difference between groups by Dunnett’s test. # Significant trend by Jonckheere’s trend test.

There were no significant increases in the total number of cells or the percentage of neutrophils, eosinophils, or lymphocytes in the BALF at week 104 (Figure 6A). The total protein and LDH activity levels in the BALF supernatant did not differ significantly from those in the control group (Figure 6B,C).

### 3.6. Concentration of N-ERC/Mesothelin in the Serum

The serum mesothelin level in mesothelioma-bearing rats was significantly higher compared to the control rats and the MWCNT-treated rats without mesothelioma (Figure 6D). No histological type-related difference in serum mesothelin was observed (Figure S3). When analyzing the data set of animals without mesothelioma, the serum mesothelin concentration was significantly higher in the high-dose group than the control group and showed a significant dose-dependent increase (Figure 6E). The data for the extra-high-dose group were excluded from Figure 6E because only two animals without mesothelioma survived to terminal necropsy.

### 3.7. Measurement of the Lung Burden and the Fiber Count in the PLF

**Figure 7:**
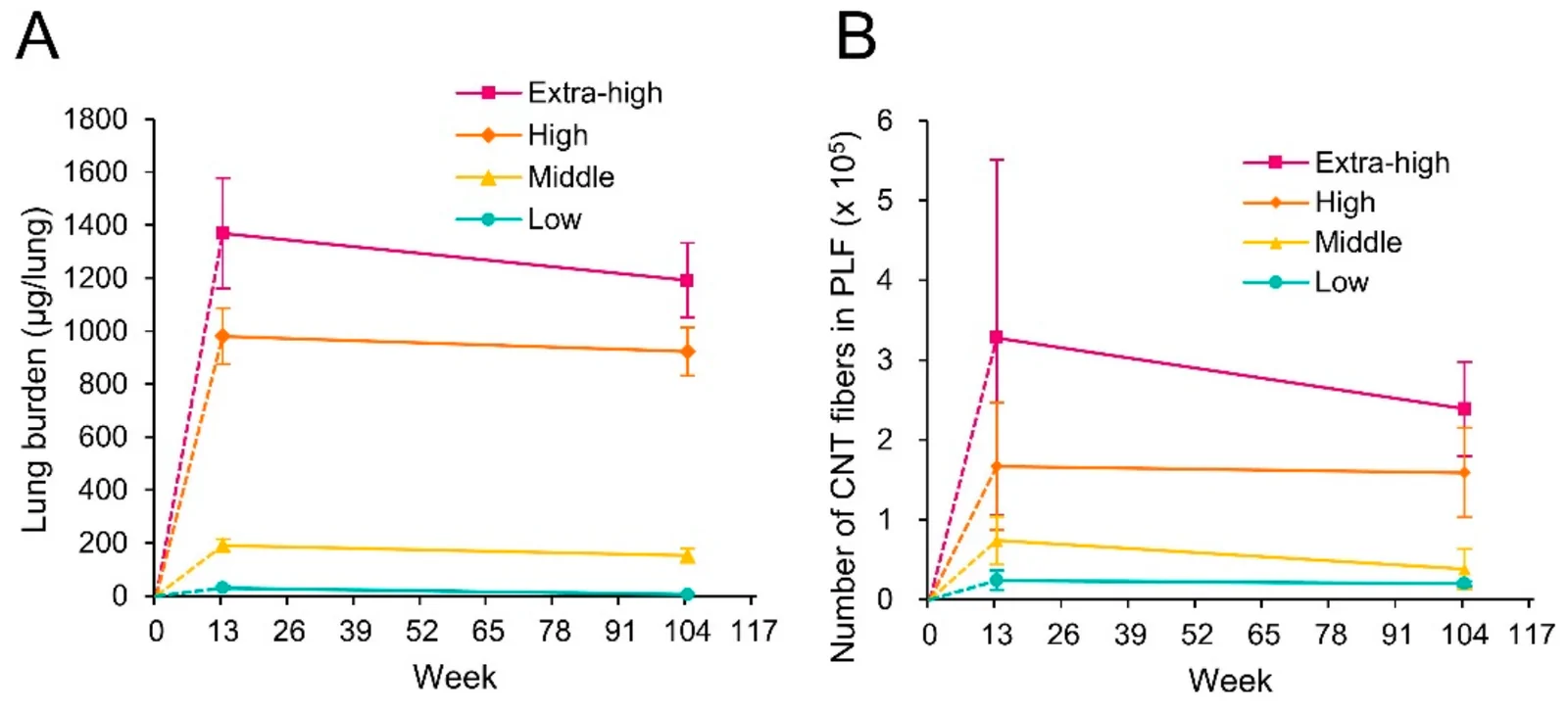
Lung burden and number of fibers in the PLF at week 13 and 104. (**A**) Lung burdens at weeks 13 and 104 (µg MWCNT-7 per lung). (**B**) Number of MWCNT-7 fibers in the PLF at weeks 13 and 104. Analyses were not performed at week 0. Dashed lines connecting zero and the data at week 13 are added for improved understanding of the time-course changes. Error bars show standard deviations.

Thirteen instillations of MWCNT-7 at 0.0175, 0.07, 0.28, and 0.42 mg/kg body weight gave a total of 0.2275, 0.91, 3.64, and 5.46 mg/kg administered into the lungs of the low-dose, middle-dose, high-dose, and extra-high-dose groups, respectively. This corresponds to 64.2, 254.5, 1,014.2, and 1,518.9 μg/rat (Table S10). The resultant lung burdens at week 13 were 30.2, 191.0, 980.4, and 1368.8 μg/rat (Table 4). Thus, the ratio of the amount of MWCNT-7 in the lung at week 13 to the total instilled dose in the low-dose, middle-dose, high-dose, and extra-high-dose groups was 47.0%, 75.0%, 96.7%, and 90.1%, respectively. At week 104, lung burdens were significantly decreased in the low-dose group, but not significantly decreased in the middle-dose, high-dose, or extra-high-dose groups compared with the peak burdens at week 13 (Table 4 and Figure 7A). The ratio of the lung burdens at week 104 to the total instilled dose in the low-dose, middle-dose, high-dose, and extra-high-dose groups was 9.7%, 60.3%, 91.0%, and 78.4%, respectively, suggesting that MWCNT-7 clearance was impaired in all groups except the low-dose group.

Table 4 summarizes the numbers of MWCNT fibers in the PLF at week 13 and week 104. Similar to the lung burden, the number of fibers increased in a dose-dependent manner at both time points (Figure 7B). The average numbers in each group showed no significant time-course change (Figure 7B).

## 4. Discussion

### 4.1. Dose-Dependent Induction of Lung and Pleural Neoplasms

In the present study, we administered MWCNT-7 intratracheally to five groups of rats (four main groups and an additional extra-high-dose group) once a week for a total of 13 weeks and observed the rats for an additional 91 weeks (104-week study period). This allowed us to obtain the dose–response relationships of two types of neoplasms, lung tumors and pleural mesothelioma, induced by MWCNT. The incidence of lung tumors (adenomas and carcinomas combined) was significantly increased in the middle-dose and the high-dose groups, and there were dose-dependent increases in broncho-alveolar adenoma and total tumors among the four main groups. In addition to bronchiolo-alveolar adenocarcinomas, two lung tumor-type adenosquamous and squamous cell carcinomas, which are extremely rare in F344 rats (0.04% each [60]), were detected in rats in the middle-dose and high-dose groups (Table 3). These uncommon neoplasms were observed only in the highest concentration groups in the inhalation study by Kasai et al. [14]: squamous cell carcinoma in 1 of 50 female rats, adenosquamous carcinoma in 1 of 50 male rats and 1 of 50 female rats. This indicates that the adenosquamous and squamous cell carcinomas identified in the present study were very likely related to the dose of MWCNT-7 administered to the rats. The no-observed-adverse-effect level (NOAEL) of lung tumor induction was 0.064 mg/rat with a peak lung burden of 30.2 µg/lung (low-dose group; 0.0175 mg/kg).

The incidence of malignant pleural mesothelioma was significantly increased in the high-dose and extra-high-dose groups and showed a clear dose-dependent increase across the treated groups. The histopathological features of the pleural mesotheliomas were largely consistent with those reported in our previous study [23], including the region susceptible to mesotheliomagenesis (mediastinum), with a predominance of sarcomatoid-type histological patterns and bone formation observed in sarcomatoid mesotheliomas. Pleural mesotheliomas were observed in two rats in the middle-dose group, but pleural mesotheliomas did not develop in the low-dose group. The extremely low spontaneous incidence of pleural mesotheliomas in F344 rats (0.03% [61]) indicates that mesothelioma development was related to MWCNT-7 treatment, so a NOAEL for pleural mesothelioma was concluded to be 0.064 mg/rat with a peak lung burden of 30.2 µg/lung (low-dose group; 0.0175 mg/kg). In previous 2-year ITI studies using MWCNT-7 (as a test material or as a positive reference material) [22,25,26], early mortality due to pleural mesothelioma often precluded the development of lung tumors, but our present experimental design allowed the evaluation of both tumor types.

### 4.2. Rationale: The 13-Week ITI Protocol Can Evaluate Lung Carcinogenicity in Rats

Our experimental design was based on three key premises. First, the chronic toxicity of MWCNT-7 can be accurately characterized by lung burden. Since the ITI, of course, differs from the inhalation in exposure route, dose-rate, deposition pattern, and clearance, the lung burden seems the only metric that can bridge both methods. Kasai and Fukushima’s review of MWCNT-7 inhalation tests they had performed (1-day, 2-week, 13-week, and 2-year durations) showed that the lung burden correlated well with the product of exposure concentration and duration (total exposure dose), and that granulomatous lesion incidence increased proportionally with the lung burden (see Figure 3 in [54]). The 2-year inhalation study demonstrated a concentration-dependent induction of lung tumors, with significantly increased incidence in the 0.2 and 2 mg/m^3^ groups, corresponding to lung burdens of 0.15 and 1.8 mg/lung, respectively. In our previous 2-year ITI study, administration of MWCNT-7 at 0.125 mg/kg and 0.5 mg/kg body weight once every 4 weeks for 2 years resulted in administration of total doses of 3.25 mg/kg and 13 mg/kg body weight, and lung burdens of 0.9 mg/lung and 3.6 mg/lung at 2 years [23]. There was a significant induction of both lung tumors and mesothelioma in the high-dose group, and 4 of 29 rats in the low-dose group also developed malignant pleural mesotheliomas. Therefore in the present study, target lung burdens of approximately 0.05 to 1.0 mg/lung in the three main groups administered MWCNT-7 were set. Rats were administered 0.0175, 0.07, and 0.28 mg/kg body weight (and 0.42 mg/kg body weight in the extra-high-dose group) once a week for 13 weeks for total doses of 0.2275 mg/kg (0.064 mg/rat), 0.91 mg/kg (0.255 mg/rat), and 3.64 mg/kg (1.014 mg/rat), and 5.46 mg/kg (1.519 mg/rat) in the extra-high-dose group, resulting in lung burdens of 0.0302, 0.191, and 0.98 mg/lung, and 1.369 mg/lung in the extra-high-dose group, after the final instillation at week 13 (Table 4 and Table S10). The dose vs. lung burden graph shown in Figure S5 indicates that for substances similar to MWCNT-7, it is possible to estimate the lung burden that will result from the total dose of material administered into the lungs of test rats and to administer appropriate doses to enable determination of the relative toxicity of the test substance based on lung burden.

Second, regarding the administration regimen, early asbestos inhalation studies by Wagner et al. and Davis et al. revealed that continuous daily exposure over 2 years was not necessarily required to evaluate asbestos-related lung carcinogenesis in rats: rather, values of total lung burdens and a long-term observation period were critical factors [62,63]. A comparative study for rat ITI technique revealed that two dosing regimens, a single administration of MWCNT or the same total dose as the single administration but divided over four administrations (every other day in a 6-day administration period), resulted in a similar level of MWCNT deposition in the lung and similar histopathological findings at days 14, 21, and 91 [55]. We previously conducted a 2-year intermittent ITI study of MWCNT-7 in rats, aiming to replicate the lung burden time-course of a 2-year daily inhalation study, and succeeded in reproducing MWCNT-7’s lung carcinogenicity [23]. The results of the 13-week administration period discussed above indicate that a similar (lung burden)/(total dose of administered MWCNT-7) ratio yielding approximately 1 mg/rat observed with the 2-year intermittent ITI administration period can be achieved with a shortened administrative period. Tumor induction was also similar in the 2-year and 13-week administration period study groups administered 1.215 and 1.014 mg/rat: 2-year administration of a total dose of MWCNT-7 of 1.215 mg/rat resulted in a lung burden of 0.9 mg/lung and lung tumors developed in 3 of 29 animals (10.3%) [23], and a 13-week administration of a total dose of MWCNT-7 of 1.014 mg/rat resulted in a lung burden of 0.98 mg/lung at 13 weeks and 0.92 mg/lung at 104 weeks and tumors developed in 7 of 40 animals (17.5%) (Table 3). Therefore, lung burden and lung tumor development resulting from the 13-week ITI administration protocol mirrored that of the 2-year inhalation and 2-year ITI administration protocols. Another important finding of the present study is that BALF analysis at the termination of the study, 91 weeks after the final MWCNT-7 administration, indicated that inflammatory parameters were not increased (Figure 6), indicating a lack of strong, acute inflammation at that time. This suggests that lung tumor occurrence could be linked to MWCNT-7-associated low-level chronic inflammation that persisted for a period of time after final instillation at week 13 and/or other unknown factors induced by the fibers retained in the lung. Furthermore, Yamano et al. suggested the importance of setting a recovery phase in rat inhalation studies for “unmasking” alveolar space-biased inflammation, which is important for evaluation of human-relevant interstitial events in rat models [64]. Altogether, it can be concluded that an administration period of 13 weeks with an observation period of 2 years is reasonable for evaluation of the carcinogenicity of MWCNT-7.

Third, it is important that the total amount of material introduced into the lung was similar to the inhalation study [14], which does not exceed the level of lung “overload” in rats. Lung overload refers to an impairment of particle clearance by macrophages caused by poorly soluble low toxicity (PSLT) particles such as TiO_2_ and carbon black, and can result in non-toxic particles inducing lung cancer in rats, as described by Morrow [65]. By definition, MWCNT-7 is not considered a PSLT particle due to its high toxicity and high aspect ratio [66]. For example, the retained amounts of MWCNT-7 that significantly increased the lung tumor incidence in the present study (0.98 mg/lung) and in the inhalation study by Kasai et al. (0.15 mg/lung) were much lower than those of the TiO_2_ (a PSLT particle) used in ITI studies (30 mg in [67] or 120 mg/lung in [68]) as well as the TiO_2_ used in 2-year inhalation studies [69,70,71] (e.g., 12 mg/lung in the top concentration group in [71]). Kasai and Fukushima examined data and tissue samples from 1-day, 2-week, 13-week, and 104-week whole body inhalation studies in which rats were exposed to aerosolized MWCNT-7 [54]. In the 2-year inhalation study, Kasai et al. did not indicate any overt differences in MWCNT-7 retention in the lung or the condition of the MWCNT-7 fibers in alveolar macrophages among the three groups (0.02, 0.2, 2 mg/m^3^ MWCNT-7), resulting in lung burdens at 2 years of 0.010, 0.152, and 1.797 mg/lung MWCNT-7 in male rats and 0.008, 0.118, and 1.154 mg/lung in female rats [14]. There was no threshold of impaired removal of fibers from the lung in any of the studies. Rather, the retention of the MWCNT-7 in the lung was clearly correlated with the number of days of exposure, even in the 2 mg/m^3^ group (see Figure 5 in the Kasai et al. study [14]). Kasai and Fukushima propose that the low clearance rate of MWCNT-7 out of the lung alveoli may be caused by pulmonary macrophage immobility resulting from other CNT-specific mechanisms [54]. Therefore, lung burdens of up to 1.8 mg/lung MWCNT-7 do not result in a lung overload-like condition. Lung burdens (per gram lung wet of weight) in the inhalation study (1.797/2.37 = 0.76 mg/g lung in the male 2 mg/m^3^ group) and the present ITI study (0.980/1.86 = 0.52 mg/g lung in the high-dose group; 1.369 mg/1.77 g = 0.77 mg/g lung in the extra-high-dose group) were indeed lower than the criteria of rat overload by PSLT particles, 1–4.5 mg/g lung [66]. As for volumetric criteria, Kasai et al. also concluded that the lung burden of everyday inhalation and even total lung burden over the 2-year inhalation were far below the lower limit of 0.1–0.3 µL/g lung tissue that is proposed by Pauluhn [43] to be the minimal volume of MWCNT associated with lung overload. Another critical point regarding high lung burdens is that high levels of MWCNT-7 can lead to persistent toxicity and chronic inflammation in the alveolar space, which can lead to rat lung carcinogenicity. While chronic inflammation associated with MWCNT-7 exposure is expected to occur in the 2-year inhalation and 2-year-ITI administration protocols, induction of tumorigenesis by extended inflammation after the termination of MWCNT-7 exposure may not always be physiologically relevant. This is basically similar to the overload induction of lung tumors [64,66,72]. Therefore, as noted above, an important aspect of the 13-week ITI administration protocol is that lung burdens were identified which resulted in induction of pulmonary tumors without concomitant inflammation at 91 weeks after the final instillation of MWCNT-7. Thus, the dose setting of instillation of MWCNT-7 in the present study was appropriate not only for comparison with the inhalation study but to allow identification of administration levels that did not result in excessive lung burdens which could lead to lung carcinogenicity by non-specific, overload-like phenomenon.

**Figure 8:**
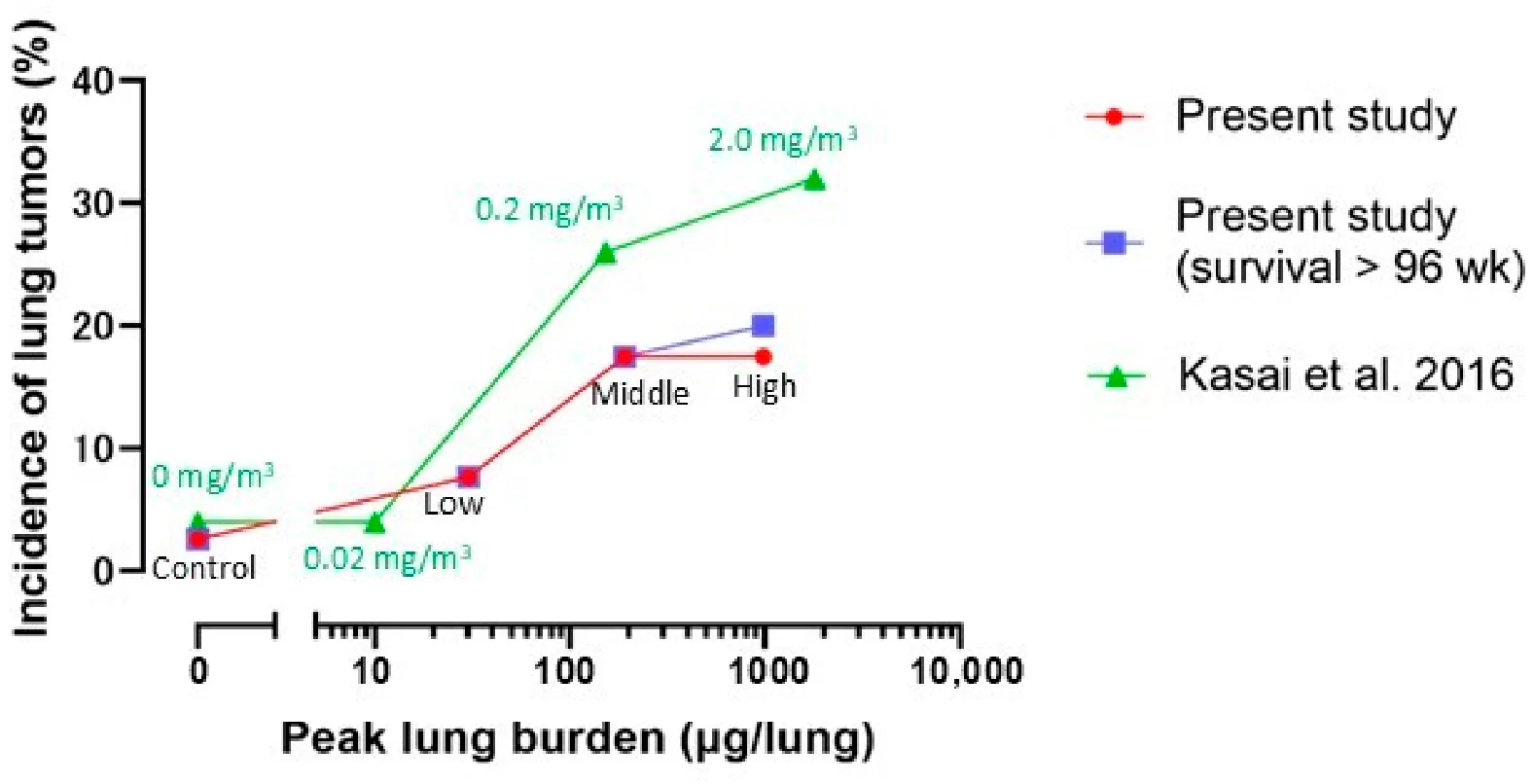
Comparison of the lung tumor inducibility between the ITI and inhalation studies. Dose–response curves of lung tumor induction based on the lung burden (peak lung burden) in the present study (red line; the present study, 4 main groups) and the inhalation study (Kasai et al., 2016 [14]; green line). The blue line is an alternative interpretation of the data: 5 rats whose lung carcinogenicity was not evaluated due to early death from pleural mesothelioma before week 96 (when the first lung tumor was observed) were excluded.

Based on these considerations, we conclude that our ITI experiment evaluates CNT lung carcinogenicity comparably to inhalation studies. The dose-dependent induction of lung tumors (total lung tumor incidence (%), adenoma + carcinoma) shows a similar dose–response relationship to the inhalation study by Kasai et al. (Figure 8) [14]. In our evaluation of lung tumors, the extra-high-dose group (about 1.5 mg/rat) was excluded. This dose was inappropriate for lung carcinogenicity evaluation because of the development of malignant pleural mesotheliomas and the resultant early deaths before lung tumors could develop in this group. Similarly, in the high-dose group (about 1 mg/rat), pleural mesotheliomas may have interfered with lung tumor assessment. When recalculating lung tumor incidence excluding rats that died or became moribund from mesothelioma before week 96 (when the first lung tumor was observed), the incidence increased slightly (Figure 8, blue line). Importantly, the Poly-3 test showed a statistically significant dose-dependent development of lung carcinogenicity when early death was considered, especially mesothelioma-related mortality (Table 3). The similarity of the dose–response curves of the 2-year inhalation study and the present ITI study was also confirmed when the lung tumor incidence (total lung tumor incidence (%), adenoma + carcinoma) was plotted against the AUC (area under the curve) of the lung burden (Figure S4). Because we measured the lung burden at only two time points, we use the word “AUC” as a relatively conceptual meaning rather than a pharmacological meaning [54,73]. Although we propose that the 13-week ITI protocol can be used to evaluate the lung carcinogenicity of MWCNT-7, as discussed earlier in this section, whether the peak burden at week 13 was more relevant to MWCNT-7 carcinogenicity than the lung burden at week 104 is still inconclusive. Lung carcinogenicity would be linked to the low-level chronic inflammation and/or other effects induced by fibers retained in the lung, as discussed above. Therefore, the AUC, the lung burden multiplied by the duration of the retention of the fibers in the lung, is likely more relevant to lung carcinogenicity than the peak lung burden at week 13 or the lung burden at termination. However, considering that clearance of CNTs was low, in particular the lung burden at week 13 was similar to that at week 104, the peak burden at week 13 seems to be a practical metric when comparing the ITI protocol with inhalation.

These findings suggest that ITI could contribute to risk characterization of CNT-induced lung carcinogenicity in the future. Although highly speculative at this time, we may be able to derive risk assessment values such as human equivalent NOAEC or BMCL_10_ values using the rat lung burden-based NOAEL or BMDL_10_ combined with adjusting parameters: alveolar epithelial surface area, inhaled air volume, and deposition rate calculated using a Multiple-Path Particle Dosimetry Model (https://www.ara.com/mppd/ (accessed on 15 July 2026)) with the data of the Mass Median Aerodynamic Diameter as shown in the inhalation study by Kasai et al. [14]. At the time of this writing, comparing ITI administration of MWCNT-7 with inhalation of MWCNT-7 is the only possible comparison since no other long-term inhalation studies with CNTs have been performed. Therefore, it remains unknown whether experimental results will support our proposal that the 13-week ITI protocol could be an alternative to the 2-year inhalation studies for risk characterization of other CNTs. Dose–response curves resulting from future ITI studies for some CNTs may possibly be shifted along the X-axis and/or Y-axis when compared with inhalation based on lung burden, while those for other CNTs might be completely different from inhalation. Thus, it is crucial to first seek the tendency or rule of relationship between ITI and inhalation by analyzing various types of CNTs. However, given that only a single 2-year inhalation study has been carried out and that performing a long-term inhalation experiment is generally highly laborious and costly, at the present time the repetitive ITI protocol appears to be an approach worthy of investigation for obtaining data that can be used in risk assessment, at least of thick, rigid CNTs (similar to MWCNT-7). It is critically important for workplace safety and the safety of users of such materials that risk assessment of CNTs is conducted, but relying on inhalation studies that are rarely, or possibly will never be, performed is unreasonable.

### 4.3. Caveats in Using the ITI Technique for Evaluation of Lung Carcinogenicity

We also note that bolus exposure by ITI cannot fully reproduce exposure by inhalation. Differences in fiber deposition and clearance compared to inhalation may influence tumor induction. First, the incidence and severity of granulomatous lesions were high in the present ITI study (Table 2). Previous studies suggest ITI induces granulomatous lesions more readily than inhalation [54,74], so a relatively larger portion of exposed CNTs may be deposited in granulomas in ITI studies. CNT fibers surrounded by granulomatous tissue are not cleared from the lung, which will contribute to the retention of CNTs in the lung (Figure 7A). Shinohara et al. found that a single intratracheal dose of short fibers was rarely eliminated from the rat lung by day 364 and they seemed to be contained in “stable” granulomas [75], and Ahmed et al. reported that about 80% of MWCNT-7 fibers remained in the lung 2 years after instillation [27]. Given that granulomatous change is a general protective response against foreign bodies entering the tissues [76,77] and that there is no location relevance between mesothelioma and granuloma lesions in rodent i.p. injection models, the CNT fibers encapsulated in granulomas are highly unlikely to contribute to tumorigenesis in the lung [18,78,79]. Thus, the susceptibility to granulomatous change after instillation of MWCNT will likely lead to relatively lower incidences of lung tumors in ITI studies compared to inhalation studies, as is shown in Figure 8 and Figure S4.

In addition, the deposition pattern of CNT fibers administered via ITI may not be uniform throughout the lung or within each lobe. Inhalation studies showed MWCNT fibers were evenly distributed across all lung lobes, including the subpleural region of the lungs [55,80,81]. In contrast, under ITI conditions, agglomerated CNT fibers tended to be retained in the terminal bronchioles [82], and even well-dispersed CNTs were localized more in centrilobular than pleural regions [83]. Comparative studies suggested that nanomaterials, including CNTs, are more evenly distributed when an aerosolizer was used instead of a feeding cannula for instillation [84,85], but even with the aerosolizers, slight non-uniform patterns remained. For example, CNTs tend to accumulate in posterior rather than anterior lung regions [55,86]. In the present study, despite using a well-dispersed sample and an aerosolizer, CNT deposition was still somewhat uneven, as evidenced by macroscopic findings in the lungs with the posterior regions being blacker (Figure 3D–F): this posterior-predominant black coloration represents the microscopic findings of MWCNT fiber deposition composed of aggregates of CNT-engulfing macrophages and isolated fibers within the alveolar space. However, whether the macroscopically uneven deposition of the administered CNT is related to the outcome of the carcinogenicity evaluation is unclear. Our thorough histological search of bronchiolo-alveolar hyperplasias in each lung lobe revealed no anterior–posterior-biased distribution of the lesions; rather, a preference of lesion development in the center to the peripheral region of the lung parenchyma was suggested (Figure S2).

The ITI experiment also induced pleural mesothelioma in rats, which is the most critical difference compared with the inhalation study by Kasai et al. [14]. Asbestos fibers that translocate into the pleural cavity are expected to evoke mesothelial cell cytotoxicity and release of a damage-associated molecular pattern (DAMP) protein, high mobility group box 1 (HMGB1), resulting in inflammation, cell transformation, and development of mesothelioma [87,88,89,90]. Rigid MWCNT fibers are expected to operate via a similar mechanism [11,26], with mesothelial cell cytotoxicity and chronic HMGB1-mediated inflammation leading to mutations in tumor suppressor genes such as CDKN2A in mesothelial cells [11,35], resulting in the development of mesothelioma. In the inhalation study, the induction of these events would be expected to be low during the first year and to increase toward the end of the study. This suggests that a portion of the mesothelial hyperplasias that developed in the inhalation study would have progressed to mesothelioma over time. It is notable that the results of the 12–24 month asbestos inhalation exposure studies, including studies using asbestos as a positive control, cited by IARC Monograph 100C were also generally negative for the induction of pleural mesotheliomas: 33 studies reported no development of pleural mesotheliomas, 10 studies reported the development of a single mesothelioma, and only 9 studies (all 12 month exposure studies) reported the development of two or more mesotheliomas (see Tables 3.1 and 3.2 in IARC Monograph 100C). Thus, the results of the MWCNT-7 inhalation exposure studies agree with the majority of asbestos inhalation exposure studies, and agree with the premise that inhalation exposure to a pleural carcinogen may not always cause pleural mesotheliomas in rats over the course of the study.

### 4.4. Duration of Fiber Exposure to the Pleura During a 2-Year Experimental Period

As noted above, one factor in the lack of pleural mesothelioma induction in the inhalation study by Kasai et al. [14] is that induction of carcinogenic pathways would be expected to be weak during the first several months of the study. In contrast to the inhalation study, in the current ITI study, maximal induction of carcinogenic events would occur at the beginning of the study, allowing time for the development of mesothelioma. The timing of mesothelioma development is also supported by the results of our previous 2-year intermittent ITI study [23] when compared to the present ITI study. In the previous ITI study, F344 male rats in the low-dose group were administered a total of 1.215 mg/rat over the course of 2 years [23]. This resulted in a lung burden of 920 µg/lung at 104 weeks (Table S11). These values are similar to those of the high-dose group in the present study: a lung burden of 980 µg/lung at the end of the 13-week administration period. Notably, the incidence of pleural mesothelioma in the previous study in the group with a lung burden of 920 µg/lung was 4/29 animals (13.8%) while in the present study the incidence of pleural mesothelioma in the group with a lung burden of 980 µg/lung was 11/40 animals (27.5%) (Table S11). Thus, when the rats were exposed to the final total dose of 1.014 mg/rat MWCNT-7 at 13 weeks with a 91 week follow-up, the incidence of mesothelioma was increased compared to the rats exposed to the final total dose of 1.215 mg/rat MWCNT-7 only at the end of the study. These findings suggest that ITI studies are superior to inhalation studies for assessment of the pleural toxicity/carcinogenicity of inhaled MWCNTs in short-lived animals such as rats.

**Figure 9:**
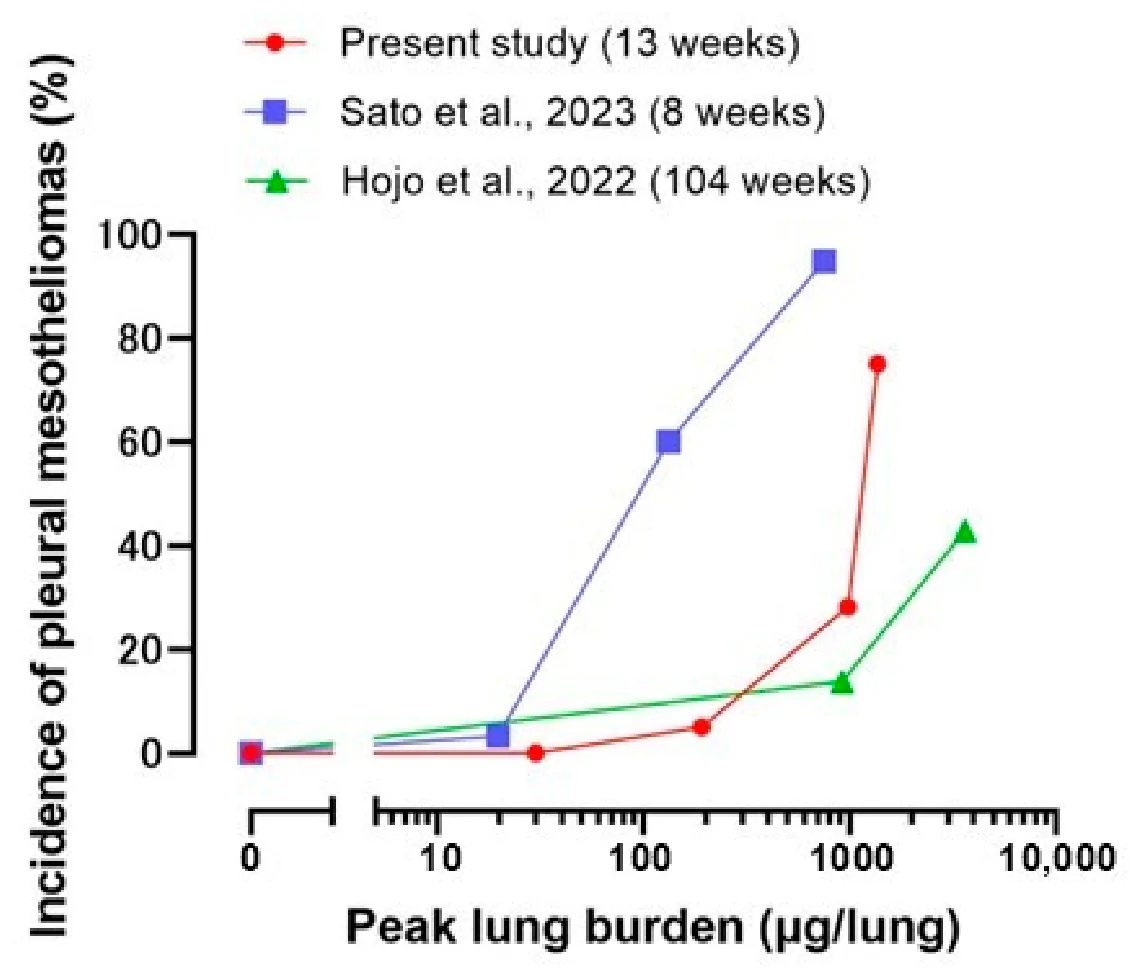
Comparison of mesothelioma inducibility among 3 ITI studies. Dose–response curves of the incidence of pleural mesothelioma obtained from 3 different 2-year ITI protocols evaluating MWCNT-7: the present study with a 13-week instillation period (red line; once a week for 13 weeks), the study by Sato et al. with an 8-week instillation period (blue line; once a week for 8 weeks, Sato et al., 2023 [25]), and the study by Hojo et al. with a 2-year intermittent instillation (green line; once every 4 weeks, 26 times, Hojo et al., 2022 [23]). The burdens in the Sato et al. study are from the data collected at week 13 (published) rather than the lung burden at week 8 (actual peak; not published).

The differences in the dosing regimen discussed above, namely earlier administration of MWCNT-7 resulting in stronger induction of carcinogenic pathways and increased time for the development of pleural mesothelioma, are also seen in other studies. Sato et al. performed a 2-year ITI test for MWCNT-7 with three dosing groups and showed a clear dose relationship in the induction of pleural mesothelioma [25]. Sato et al. set the administration period as 8 weeks at the beginning of the experiment, and compared with the present study, their experiment resulted in a higher incidence of mesothelioma (Figure 9). Thus, when comparing 8-week administration, 13-week administration, and 2-year administration periods that include more than two dose groups in 2-year ITI experiments, the dose–response curve for mesothelioma induction becomes steeper as the administration period shortens (Figure 9). Therefore, setting a longer administration period to reduce the administered dose per instillation and/or keep long term clearance periods between each instillation may reduce the development of pleural mesothelioma during the study period. However, long-term intermittent instillation (e.g., 2 years) is labor-intensive, making it more difficult to test a large number of materials. Importantly, as discussed earlier, the 13-week administration regimen used in this study can achieve similar levels of lung burdens to the inhalation study and give a long-term observation period (i.e., sufficient to develop lung and pleural tumors). Therefore, the 13-week administration period appears to be a well-balanced experimental design that evaluates two types of neoplasms.

In the present study, we set an extra-high-dose group (1.5 mg/rat) to confirm the upper dose limit of MWCNT-7 in ITI experiments. In a previous 2-year ITI study by Numano et al., 1.5 mg of MWCNT-7 was administered over 12 weeks [22]. In their study, 95% of the rats died from mesothelioma before lung tumors could develop. We speculate that the relatively lower incidence (70%) in the extra-high-dose group of the present study may be related to the difference in dose and volume of each administration. Numano et al. used a fixed dose for all rats over 12 weeks (a total dose of 1.5 mg/rat was simply divided into 12 aliquots) [22], while we set doses kilogram per body weight for each individual animal (totaling about 1.5 mg/rat over 13 weeks; see Table S10). The fixed dose likely placed a heavier burden on younger rats. The fixed dose volume in the study by Numano et al. (0.5 mL/rat) was also relatively higher compared to our per kilogram setting (1 mL/kg, or 0.25 mL/250g rat). These differences in the dosing regimens may have led to the difference in mesothelioma incidence. While the incidence of mesothelioma was slightly lower in our study, the development of mesotheliomas interfered with lung carcinogenicity evaluation. In addition, as shown in Figure 9, the response of the extra-high-dose group to the high lung burden was not similar to the 2-year administration regimen. This indicates that the high-dose group of about 1 mg/rat is a reasonable upper dose for assessment of MWCNT-7 lung and pleural carcinogenicity. The dose–response curve for the present ITI study shown in Figure 9 also suggests that in future studies assessing the lung and pleural carcinogenicity of other MWCNTs, the use of “extra-high-dose” groups may help to identify the doses that are appropriate for evaluating the carcinogenicity of the tested MWCNTs for determination of rat NOAEL or BMDL_10_ values.

### 4.5. Fiber Translocation to the Pleural Cavity and Postulated Pathogenesis of Pleural Mesothelioma in Rats Administered MWCNT by Repetitive ITI

In addition to differences in the induction of pleural mesothelioma, discussed above, a second important difference between our ITI studies and the 2-year inhalation study by Kasai et al. [14] is the enormous elevation of free MWCNT-7 fibers in the PLF in the 13-week and 2-year ITI studies compared with the 2-year inhalation study (Table S11). In the 2-year inhalation study, exposure to 0.02, 0.2 and 2 mg/m^3^ MWCNT-7 for 2 years resulted in lung burdens of 10, 152, and 1798 µg/lung in male F344 rats at 104 weeks. In these animals, at 104 weeks, 38, 134, and 1468 MWCNT-7 fibers were found in the pleural lavage. In the current ITI study, administration of 0.0175, 0.07, and 0.28 mg/kg body weight MWCNT-7 over the course of 13 weeks to male F344 rats resulted in lung burdens of 30, 191, and 980 µg/lung at the end of the 13-week administration period and 6, 154, and 923 µg/lung at 104 weeks. In these animals, 24,210, 74,314, and 166,528 MWCNT-7 fibers were found in the PLF at 13 weeks and 20,448, 38,122, and 158,563 MWCNT-7 fibers were found in the PLF at 104 weeks. Notably, there is a technical difference in the preparation of the PLF sample for fiber counting between the present study and Kasai et al.’s study: we collected fibers from the PLF at a stronger centrifuge condition (20,000× *g*) than in the study by Kasai et al. (3000× *g*), which may affect the results of fiber counting. However, another 2-year ITI study by Sato et al. indicated that the number of MWCNT-7 fibers in the PLF was 1–2 orders of magnitude higher than in the inhalation study, and their experimental conditions for the preparation of the PLF sample were identical to that of the inhalation study [25]. Thus, the repetitive ITI technique may indeed be related to an increase in free MWCNT-7 fibers in the pleural cavity. Since the number of MWCNT-7 fibers in the PLF in the highest concentration group in the inhalation study (1468) is one order of magnitude lower than the NOAEL group in the present study (20,448) (Table S11), it seems reasonable that pleural mesothelioma did not occur in the inhalation study.

A possible factor in the elevation of free MWCNT-7 fibers in the PLF in the 13-week and 2-year ITI studies compared with the 2-year inhalation study may concern the translocation pathways from the lung into the pleural cavity. The translocation pathways of MWCNT fibers from the lung alveoli into the pleural cavity have not yet been established; however, in both humans and animals it has been suggested that thick, rigid MWCNT fibers can move from the lung into the pleural cavity via the lymphatic system or via direct penetration of the visceral pleura as has been suggested for asbestos [23,91,92,93,94]. We infer that bolus exposure promotes CNT penetration of the visceral pleura because microscopic/SEM findings of CNT fibers penetrating into visceral pleura have only been reported in studies using ITI or pharyngeal aspiration [23,81,95]. Another proposed route of translocation of asbestos from the lung into the pleural cavity is via the lymphatics. One hypothesis is that asbestos fibers in subpleural lymphatics penetrate through the wall of subpleural lymphatic vessels and translocate into the visceral pleura [94]. Another hypothesis is that a proportion of asbestos fibers are cleared from the lung via pulmonary lymphatics and then translocate from pulmonary lymphatics into blood capillaries. These fibers then secondarily follow the pressure gradient from systemic blood capillaries into the fluid that continuously moves out of systemic capillaries in the parietal pleura and into the pleural cavity [91,94]. Similarly to these proposals, it is well known that particles instilled into the lung can translocate into the pulmonary lymphatics [94,96]. Also, similarly to asbestos, which can be found in numerous extra-pulmonary organs in subjects exposed to even low amounts of asbestos [97,98], after inhalation exposure to MWCNT-7, fibers can also be found in a variety of extra-pulmonary organs [14,99]. Therefore, the proposed pathways by which asbestos translocates from the lung into the pleural cavity also have the potential to translocate thick, rigid MWCNT fibers into the pleural cavity. Possible effects of administration of very large amounts of MWCNT-7 into the lung using instillation on lung-to-pleura translocation pathways needs to be investigated in future studies.

Even if the bolus effect of ITI artificially enhances fiber translocation into the pleural cavity, this does not mean that intratracheal administration causes nonspecific translocation into the pleural cavity. Rather, the shape of CNT fibers may be linked to a critical step—translocation to the pleural cavity—which specifies mesothelioma induction in the rat ITI model. After instillation into the lung thick, rigid CNTs like MWCNT-7 (diameter 80–90 nm) and MWCNT-N (diameter 60 nm) [21] induced high incidences of pleural mesotheliomas, while much thicker CNTs like MWCNT-A (diameter 150 nm) and VGCF-H (diameter 148 nm) rarely induced development of pleural mesotheliomas: 0/20 in males administered MWCNT-A at 1 mg/rat; 2/30 in males and 0/30 in females administered VGCF-H at approximately 0.8 mg/rat [24,25]. The 2-year study by Sato et al. [25], administered MWCNT-7 and VGCF-H via instillation into the lung once a week for 8 weeks. At week 13, the number of VGCF-H fibers in the PLF of male rats was 15–38 times lower than the number of MWCNT-7 fibers at the corresponding doses. Due to differences in fiber size, MWCNT-7 samples had twice as many fibers per gram as VGCF-H (11 × 10^12^ vs. 6.1 × 10^12^; calculated based on the data of fiber characterization by Sato et al.). Thus, VGCF-H fibers in the PLF were approximately 7–19 times lower than MWCNT-7 fibers based on the fiber number instilled into the lung, indicating less translocation of VGCF-H fibers into the pleural cavity. In other studies, thin, tangled CNTs (e.g., SWCNT, DWCNT, and MWCNT-B) did not induce pleural mesotheliomas in 2-year experiments [24,26,27] [Naiki-Ito et al., under review; Particle Fibre Toxicology], and histology and analyses of the PLF indicated that thin, tangled DWCNT and MWCNT-S fibers did not translocate into the pleural cavity [26,27,28,95,100]. Therefore, while instillation may enhance translocation of MWCNTs into the pleural cavity after administration into the lung, the translocation of MWCNT-7 into the pleural cavity in the present ITI study can be considered to be physiologically relevant, i.e., instillation of MWCNT fibers into the lung does not cause unconditional, non-specific translocation of the fibers from the lung into the pleural cavity.

Although pleural mesothelioma is only induced in ITI experiments, the induction of mesothelioma in rat ITI experiments is an important factor for human risk assessment. Kasai et al.’s study [14] showed a dose-dependent increase in mesothelial hyperplasia, and, as discussed above, most asbestos inhalation studies did not report induction of pleural mesotheliomas. Thus, while both CNTs and asbestos translocate to the pleural cavity in rats causing mesothelial pathogenesis in inhalation experiments, tumor induction is not consistent between studies. In addition, in humans, inhaled asbestos fibers are thought to translocate to the pleural cavity over decades, making it difficult to reproduce this process and mesothelioma induction within a 2-year rodent experiment. For human exposure scenarios, how to interpret rat inhalation tests for extrapolation to the human lifetime is an unresolved question [101,102]. Currently, ITI experiments appear to be sensitive for assessment of the pleural toxicity/carcinogenicity of inhaled MWCNTs in short-lived animals such as rats. However, finding of the specific pathways by which thick, rigid MWCNT fibers would be able to translocate from the lung into the pleural cavity in humans requires further study [103]. Also, the reason why the number of fibers in the PLF at weeks 13 and 104 were similar remains unclear. It is possibly because the clearance rate of the fibers that translocated from the lung into the pleural cavity during the 13-week administrative period was low over the subsequent 91 weeks or the number of fibers in the pleural cavity was static due to the translocation of fibers from the lung into the pleural cavity and removal of fibers out of the pleural cavity being similar. Further time-course and quantitative analyses are required.

### 4.6. A Possible Serum Marker of Rat Pleural Mesothelioma

We found that the human mesothelioma marker N-ERC/mesothelin [104] may be a useful biomarker for detecting pleural mesothelioma development in MWCNT-exposed rats. While a few previous studies measured mesothelin levels in rat blood exposed to CNTs or asbestos in peritoneal injection models [105,106], the present study is the first study to evaluate serum N-ERC/mesothelin in an ITI experiment with MWCNT. While our data is biased toward advanced cases in the present study, additional data including a wider variety of mesothelioma severity should be examined in the future. Time-course analysis of the serum N-ERC level after administration of carcinogenic doses of the test MWNCT is also needed to clarify the sensitivity and specificity of N-ERC as an early detection marker (e.g., 26–52 weeks). Nevertheless, the dose-dependent response in serum levels of N-ERC/mesothelin in rats without mesothelioma (Figure 6E) suggests it can detect subtle changes in mesothelial cell status after MWCNT-7 exposure. Thus, the serum level of N-ERC has potential as a marker to help in the detection of pleural mesothelioma induction by newly developed CNTs in subchronic studies using rats.

## 5. Conclusions

MWCNT-7 was administered to male F344 rats via intratracheal instillation once a week for 13 weeks, followed by a 91-week observation period. Significant increases in lung tumors and pleural mesothelioma were observed in groups with total lung burdens equal to or greater than 0.191 mg/lung. The dose–response curve of lung burden vs. lung tumor induction was similar to that observed in the 2-year inhalation study by Kasai et al., and the NOAEL of lung tumor induction using lung burden in this study was comparable to that of the NOAEC group in the inhalation study. Pleural mesothelioma was induced by MWCNT-7, consistent with previous ITI studies. The induction and incidence of mesothelioma appear to be associated with the bolus exposure method (i.e., ITI-specific), and may also be influenced by the dosing regimen.

This study proposes a possible application of the 13-week rat ITI administration regimen for risk characterization of CNTs, in addition to hazard identification. Given that conducting OECD TG451-based 2-year inhalation studies is labor intensive and limited by the high cost of such studies, ITI studies may serve as an alternative if further comparative data from 2-year inhalation and ITI studies justify the use of ITI studies for obtaining relevant data. Furthermore, 2-year ITI studies may be valuable for investigating the carcinogenic mechanisms by which inhaled CNTs induce lung cancer and pleural mesothelioma.

## Figures and Tables

**Table 1:** Survival, body weights, and lung weights at week 104.

	Control	Low	Middle	High	Ex-High
	0 mg/kg	0.0175 mg/kg	0.07 mg/kg	0.28 mg/kg	0.42 mg/kg
Number of rats examined	38	39	40	40	20
Survival animal number (rate; %)	29 (76.3)	26 (66.7)	33 (82.5)	14 (35.0) *	3 (15.0) *
Average survival time (weeks)	100.1	99.7	102.2	93.6	90.3
Body weight (g)	381.9 ± 30	382.5 ± 27	383.6 ± 43	346.3 ± 32 *	393.1 ± 13
Absolute lung weight (g)	1.24 ± 0.15	1.19 ± 0.09	1.31 ± 0.14	1.86 ± 0.67 *	1.77 ± 0.13
Relative lung weight (g/100 g body weight)	0.31 ± 0.05	0.31 ± 0.02	0.34 ± 0.03	0.41 ± 0.03 *	0.47 ± 0.09 *
Absolute lung weight of left lobe (g)	0.43 ± 0.03	0.42 ± 0.03	0.49 ± 0.07 *	0.55 ± 0.04 *	NA
Relative lung weight of left lobe (g/100 g body weight)	0.11 ± 0.01	0.11 ± 0.01	0.12 ± 0.02	0.16 ± 0.01 *	NA

Weights: Mean ± SD. * Significantly different from Control (by Dunnett’s test). NA: not available.

**Table 2:** Histological analysis of non-neoplastic lesions in the lung and pleura.

		Control	Low	Middle	High	Ex-High
		0 mg/kg	0.0175 mg/kg	0.07 mg/kg	0.28 mg/kg	0.42 mg/kg
	Number of rats examined	38	36	39	39	19
Lung	Accumulation, alveolar macrophage	4	31 *	39 *	39 *	19 *
		[0.01]	[0.26 *]	[1.07 *]	[2.27 *]	[2.54 *]
	Granulomatous change	1	32 *	39 *	39 *	19 *
		[0.01]	[0.57 *]	[0.90 *]	[2.10 *]	[2.48 *]
	Focal fibrosis, alveolar wall	0	4	6 *	27 *	17 *
		[0]	[0.02]	[0.03 *]	[0.22 *]	[0.64 *]
	Bronchiolar hyperplasia (bronchiolization)	0	0	0	12 *	7 *
		[0]	[0]	[0]	[0.46 *]	[0.38 *]
	Bronchiolo-alveolar hyperplasia	5	8	18 *	15 *	6
		[0.02]	[0.04]	[0.12 *]	[0.10 *]	[0.07]
	Atypical hyperplasia	0	0	0	1	1
		[0]	[0]	[0]	[0.03]	[0.05]
Pleura	Visceral, focal fibrosis	0	4	21 *	38 *	19 *
		[0]	[0.02]	[0.10 *]	[0.71 *]	[0.97 *]
	Parietal, focal fibrosis	0	12 *	34 *	32 *	9 *
		[0]	[0.18 *]	[0.58 *]	[0.64 *]	[0.39 *]
	Visceral, mesothelial hyperplasia	0	0	2	11 *	13 *
		[0]	[0]	[0.01]	[0.09 *]	[0.33 *]
	Parietal, mesothelial hyperplasia	0	0	7 *	5 *	3 *
		[0]	[0]	[0.08 *]	[0.08 *]	[0.13 *]

Values show the number of animals bearing lesions. Values in square brackets are the average severity grades in all examined animals; (grade × number of animals with grade) ÷ (number of animals examined). Grade: 0, no/minimal change; 1, slight; 2, moderate; 3, marked. Animals bearing severe large granular lymphocytic leukemia and severe postmortem changes were excluded (3, 1, 1, and 1 animal(s)) in the low-dose, middle-dose, high-dose, and ex-high-dose groups, respectively). * Significantly different from the corresponding control group (incidence, Fisher’s exact test; severity score, Steel’s test).

**Table 3:** Incidence of lung tumors and malignant pleural mesothelioma.

		Control	Low	Middle	High	Ex-High
		0 mg/kg	0.0175 mg/kg	0.07 mg/kg	0.28 mg/kg	0.42 mg/kg
Number of rats examined		38	39	40	40	20
Lung	Bronchiolo-alveolar adenoma (%)	0	2 (5.1)	3 (7.5)	4 (10) ^†^	2 (10)
	Bronchiolo-alveolar adenocarcinoma (%)	1 (2.6)	1 (2.6)	4 (10.0)	2 (5.0)	1 (5.0)
	Adenosquamous carcinoma(%)	0	0	1 (2.5)	0	0
	Squamous cell carcinoma (%)	0	0	0	1 (2.5)	0
	Total lung tumors (%) (adenoma and/or carcinoma)	1 (2.6)	3 (7.7)	7 (17.5) *	7 (17.5) *^†^	3 (15)
Pleura	Malignant pleural mesothelioma (%)	0	0	2(5.0)	11 (27.5) *^†^	14 (70) *

*; Significantly different from Control (by Fisher’s exact test). ^†^; Significantly different among the main 4 groups, control, low-dose, middle-dose, and high-dose groups (by Poly-3 test).

**Table 4:** Lung burdens and number of MWCNT fibers in the PLF.

		Low	Middle	High	Ex-High
		0.0175 mg/kg	0.07 mg/kg	0.28 mg/kg	0.42 mg/kg
Lung burden (μg/lung)	week 13	30.2 ± 7	191.0 ± 35	980.4 ± 170	1368.8 ± 310
	week 104	6.2 ± 3 *	153.5 ± 32	922.6 ± 120	1191.2 ± 190
Number of MWCNT fibers in the PLF	week 13	24,210 ± 12,000	74,314 ± 31,000	166,528 ± 76,000	327,714 ± 220,000
	week 104	20,448 ± 3500	38,122 ± 25,000	158,563 ± 52,000	238,594 ± 59,000

Values: Mean ± SD. * Significantly different from week 13 (by Student’s *t*-test).

## Data Availability

The original contributions presented in this study are included in the article/Supplementary Materials. Further inquiries can be directed to the corresponding authors.

## Supplementary Materials

The following supporting information can be downloaded at: https://www.mdpi.com/article/10.3390/nano16161039/s1, Figure S1 Characterization of bulk MWCNT-7. (A) SEM image of bulk MWCNT-7. (B) Morphology of the individual fibers. Singular, straight-type fibers account for 91.8% of the total examined. The content of aggregated fibers (8.2%) was significantly higher than the Taquann-MWCNT-7 (see Figure 1B). (C,D) Distribution of fiber length and diameter of bulk MWCNT-7. This shows Taquann treatment changed neither the length nor diameter of the bulk MWCNT-7 (compare to Figure 1C,D). Figure S2 Histological distribution of bronchiolo-alveolar hyperplasia. (A) Definition of the histological area. The lung was divided into three histological regions: (1) the central region, comprised of bronchi (red), (2) the proximal acinus, comprised of terminal bronchioles and alveolar ducts (yellow), and (3) the distal acinus region, comprised of all other parenchymal regions, mainly alveoli (blue). (B) The bars represent the numbers of hyperplastic lesions found in each histological region. The numbers within the bars represent percentages. The bars are the sums of the lesions found among all examined animals in each group (40 animals per group in the main 4 groups; 5 lobes per animal; see Table S1). In the control and low-dose groups, the hyperplastic lesions were observed exclusively within the acinus regions. In contrast, in the middle-dose and high-dose groups, they were detected in the acinus regions and also in the central region. (C) Representative histological images of the lesions found in each region. B; bronchiole, TB; terminal bronchiole, AD; alveolar duct, Al; alveoli, Pl; pleura. Figure S3 Comparison of the serum mesothelin levels amongst mesothelioma-bearing rats with different histological types. The individual data shown in Figure 6D were reanalyzed based on histological diagnosis. There were no significant differences in the mesothelin concentration between different histological types. Figure S4 Comparison of the lung tumor inducibility between the ITI study and the inhalation study based on the AUC of the lung burdens. Dose-response curves were redrawn using the metric, area under the curve (AUC) of the lung burden (peak lung burden): duration (μg) × dose (day). The AUCs in the present study were derived from a simple geometric calculation using the same data shown in Figure 8 while those in the inhalation study were obtained from a previous study by Kasai and Fukushima, 2023 [54]. Figure S5 Correlation of the total dose and peak lung burden at week 13. Table S1 The disposition of the animals in this study. (a) Two rats died from choking on pelleted food and were excluded (at week 70 and 84). (b) One rat died from choking on pelleted food and was excluded (at week 69). (c) These rats were only used for histology. (d) One satellite-group-rat died from choking on pelleted food and was excluded (at week 80). (e) One satellite-group-rat died from large granular lymphocyte leukemia and was excluded (at week 83). Table S2 Quantitative analysis of metal impurities in the Taquann-MWCNT-7 by ICP-MS. Values in the parentheses were limit of quantification ND: not detected, less than limit of quantification. Table S3 Deaths prior to termination at 104 weeks among animals used in the carcinogenicity study. Table S4 Major organ weights. Weights: Mean ± SD * Significantly different from Control (by Dunnett’s test). Spleen: one rat from the middle-dose group and one rat from the high-dose group bearing large granular lymphocyte leukemia were excluded. Liver: one rat from the middle-dose group and one rat from the high-dose group bearing large granular lymphocyte leukemia were excluded. Left kidney: one rat from the middle-dose group and one rat from the high-dose group bearing kidney tumor were excluded. Right adrenal: one rat bearing severe pheochromocytoma in the middle-dose group was excluded. Table S5 Hematological analyses of rats at the terminal necropsy. Mean ± SD. WBC, white blood cell count; RBC, red blood cell count; HGB, hemoglobin concentration; HCT, hematocrit level; MCV, mean corpuscular volume; MCH, mean corpuscular hemoglobin; MCHC, mean corpuscular hemoglobin concentration; PLT, platelet count. (1) One rat bearing large granular lymphocyte leukemia in the middle-dose group was excluded. (2) One rat bearing large granular lymphocyte leukemia in the high-dose group was excluded. Table S6 List of all lung tumors found in this study. BAA: Bronchiolo-alveolar adenoma, BAC: Bronchiolo-alveolar adenocarcinoma, ASC: Adenosquamous carcinoma, SCC: Squamous cell carcinoma. * Two tumors were found in the same animal. Table S7 List of animals bearing proliferative lesions in the pleura in this study. * Diameter of the tumor nodule: small, around 1 mm; middle, around 5mm; large, more than 10 mm. Table S8 Incidences of other tumors. Table S9 Anatomical distribution of bronchiolo-alveolar hyperplasia. Table S10 Mean doses for each administration (μg/rat). The dose was set corresponding to the individual body weight of each animal for every administration. Table S11 Comparison of the mesothelial lesions between the inhalation study and ITI studies based on the lung and pleural burdens. * numbers of animal with hyperplasias in the visceral or parietal pleura.

## Abbreviations

AUC: Area under the curve; BALF: Bronchoalveolar lavage fluid; BALT: Bronchiolar-associated lymphatic tissue; B(ghi)P: Benzo[ghi]perylene; LDH: Lactate dehydrogenase; MWCNT: Multiwalled carbon nanotube; PLF: Pleural lavage fluid; RPF: Retrocardiac pleural fold; PSLT: poorly soluble low toxicity; SEM: Scanning electron microscope; UHPLC: Ultra High Performance Liquid Chromatography.
