# Supplementary material for: Dose–Response Relationships for Lung Tumor and Pleural Mesothelioma Induction by Repetitive Intratracheal Instillation of a Multiwalled Carbon Nanotube, MWCNT-7, in Rats: Comparison with Inhalation Carcinogenicity Based on Lung Burden
Source: Nanomaterials (Basel). 2026 Aug 20;16(16):1039. doi: 10.3390/nano16161039 (PMC13515796; doi:10.3390/nano16161039)
Supplement: Supplementary file 1 [file nanomaterials-16-01039-s001.zip › nanomaterials-4473910-supplementary-tables.pdf]

Table S1. The disposition of the animals in this study

<table>
<tr><th rowspan="2">Time</th><th rowspan="2" colspan="2">Group</th><th colspan="2">Control</th><th colspan="2">Low-dose</th><th colspan="2">Middle-dose</th><th colspan="2">High-dose</th><th colspan="2">Extra-High-dose</th><th rowspan="2">Item</th></tr>
<tr><th>planned</th><th>actual</th><th>planned</th><th>actual</th><th>planned</th><th>actual</th><th>planned</th><th>actual</th><th>planned</th><th>actual</th></tr>
<tr><td>Week 13</td><td colspan="2">Satellite</td><td>-</td><td>-</td><td>5</td><td>5</td><td>5</td><td>5</td><td>5</td><td>5</td><td>5</td><td>5</td><td>Lung burden, PLF</td></tr>
<tr><td rowspan="3">Until terminal necropsy (week 104)</td><td rowspan="2">Pathology for the carcinogenicity assessment</td><td>Sacrifice before the termination (humane end point or dead)</td><td rowspan="2">40</td><td>9[a)]</td><td rowspan="2">40</td><td>13[b)]</td><td rowspan="2">40</td><td>7</td><td rowspan="2">40</td><td>26</td><td rowspan="2">20</td><td>17</td><td>Histology</td></tr>
<tr><td>Terminal sacrifice wk 104</td><td>29</td><td>26</td><td>33</td><td>14</td><td>3[c)]</td><td>Histology (all)
BALF, Organ weight, Hematology
(8 animals each group)</td></tr>
<tr><td>Satellite</td><td>Terminal sacrifice wk 104</td><td>-</td><td>-</td><td>5</td><td>5</td><td>5</td><td>4[d)]</td><td>5</td><td>5</td><td>5</td><td>4[e)]</td><td>Lung burden, PLF</td></tr>
</table>

a) Two rats died from choking on pelleted food and were excluded (at week 70 and 84).

b) One rat died from choking on pelleted food and was excluded (at week 69).

c) These rats were only used for histology.

d) One satellite-group-rat died from choking on pelleted food and was excluded (at week 80).

e) One satellite-group-rat died from large granular lymphocyte leukemia and was excluded (at week 83).

Table S2. Quantitative analysis of metal impurities in the Taquann-MWCNT-7 by ICP-MS

	Al<br>(0.1)	Cr<br>(0.01)	Mn<br>(0.01)	Fe<br>(0.1)	Ni<br>(0.01)	Cu<br>(0.01)	Zn<br>(0.02)	Mo<br>(0.01)	Ba<br>(0.01)	Pb<br>(0.01)
(μg/mgCNT)	ND	0.19	0.05	5.6	0.02	0.01	0.04	ND	ND	ND

Values in the parentheses were limit of quantification

ND: not detected, less than limit of quantification

Table S3. Deaths prior to termination at 104 weeks among animals used in the carcinogenicity study

Group	Condition	Time of death (week)	Cause of death	Lung tumor
Control	Sacrificed	79	Pharinx, papilloma	
	Sacrificed	83	Pituitary gland, paris distalis, adenoma	
	Sacrificed	85	Mammary gland, fibroma	
	Sacrificed	89	Pituitary gland, paris distalis, adenoma	
	Sacrificed	91	Mammary gland, fibroma	
	Sacrificed	100	Zymbal's gland, carcinoma	
	Sacrificed	102	Large granular lymphocyte leukemia	
	Sacrificed	102	Large granular lymphocyte leukemia	
Low-dose	Sacrificed	73	Brain, high grade malignant astrocytoma	
	Sacrificed	82	Skin, hemangiosarcoma	
	Sacrificed	82	Peritoneal mesothelioma	
	Sacrificed	85	Large granular lymphocyte leukemia	
	Dead	87	Large granular lymphocyte leukemia	
	Dead	91	Large granular lymphocyte leukemia	
	Dead	96	Large granular lymphocyte leukemia	
	Sacrificed	97	Large granular lymphocyte leukemia	
	Sacrificed	97	Thymic lymphoma	
	Sacrificed	97	Pituitary gland, paris distalis, adenoma	
	Sacrificed	98	Pituitary gland, paris distalis, adenoma	
	Sacrificed	100	Femur, hemagiosarcoma	
	Sacrificed	101	Large granular lymphocyte leukemia + Peritoneal mesothelioma	+ (adenoma)
Middle-dose	Sacrificed	80	Preputial gland, carcinoma	
	Sacrificed	87	Large granular lymphocyte leukemia	
	Sacrificed	95	Pituitary gland, paris distalis, adenoma	
	Sacrificed	96	Oral cavity, amelanotic melanoma, malignat	
	Sacrificed	97	Peritoneal mesothelioma	
	Sacrificed	99	Kidney, renal cell caricinoma	
	Dead	100	Thyroid, c-cell carcinoma	
High-dose	Sacrificed	51	Brain, high grade malignant oligodendrogrlioma	
	Sacrificed	70	Large granular lymphocyte leukemia	
	Dead	77	Pleural mesothelioma	
	Sacrificed	78	Pituitary gland, paris distalis, adenoma	
	Sacrificed	78	Large granular lymphocyte leukemia	
	Sacrificed	79	Peritoneal mesothelioma	
	Sacrificed	80	Skin, hemangiosarcoma	
	Sacrificed	83	Mammary gland, fibroma	
	Sacrificed	83	Mammary gland, fibroma	
	Sacrificed	87	Pleural mesothelioma	
	Sacrificed	89	Thyroid, c-cell adenoma	
	Sacrificed	90	Pleural mesothelioma	
	Sacrificed	91	Thyroid, c-cell carcinoma	
	Sacrificed	91	Pleural mesothelioma	
	Sacrificed	91	Skin, basal cell carcinoma	
	Sacrificed	92	Skin/Subcutaneous, schwannoma, malignant	
	Sacrificed	93	Mammary gland, fibroma	
	Sacrificed	94	Pleural mesothelioma	
	Sacrificed	96	Pleural mesothelioma	
	Sacrificed	96	Large granular lymphocyte leukemia	+ (adenoma)
	Sacrificed	96	Large granular lymphocyte leukemia	
	Sacrificed	97	Intramuscular hematoma	+ (squamous cell carcinoma)
	Sacrificed	98	Large granular lymphocyte leukemia	
	Sacrificed	98	Pleural mesothelioma	+ (adenoma)
	Sacrificed	99	Pleural mesothelioma	
	Sacrificed	101	Pleural mesothelioma	
Ex-High-dose	Sacrificed	61	Pituitary gland, paris distalis, adenoma	
	Sacrificed	73	Pleural mesothelioma	
	Sacrificed	77	Pleural mesothelioma	
	Sacrificed	79	Pleural mesothelioma	
	Sacrificed	82	Large granular lymphocyte leukemia	
	Sacrificed	82	Pleural mesothelioma	
	Sacrificed	85	Pleural mesothelioma	
	Sacrificed	88	Pleural mesothelioma	
	Sacrificed	89	Peritoneal mesothelioma	
	Sacrificed	92	Pleural mesothelioma	
	Sacrificed	93	Large granular lymphocyte leukemia	
	Sacrificed	97	Pleural mesothelioma	
	Dead	97	Pleural mesothelioma	
	Sacrificed	97	Pituitary gland, paris distalis, adenoma	
	Sacrificed	98	Peritoneal mesothelioma	
	Sacrificed	101	Pleural mesothelioma	+ (adenoma)
	Sacrificed	102	Pleural mesothelioma	

Table S4. Major organ weights

Group	Control	Low	Middle	High	Ex-High
Numbers of animals	8	8	8	8	3
Brain (g)	2.23 ± 0.04	2.15 ± 0.04*	2.21 ± 0.06	2.17 ± 0.06	2.27 ± 0.08
Spleen (g)	1.08 ± 0.19	1.16 ± 0.38	1.12 ± 0.03	1.17 ± 0.29	1.06 ± 0.1
Liver (g)	12.1 ± 1.04	11.9 ± 2.17	12.7 ± 0.89	13.3 ± 1.48	12.7 ± 0.57
Heart (g)	1.02 ± 0.06	1.02 ± 0.03	1.05 ± 0.07	1.05 ± 0.07	1.04 ± 0.1
Left kidney (g)	1.4 ± 0.06	1.32 ± 0.13	1.42 ± 0.1	1.41 ± 0.16	1.48 ± 0.08
Right kidney (g)	1.38 ± 0.09	1.32 ± 0.13	1.39 ± 0.1	1.42 ± 0.13	1.42 ± 0.12
Left adrenal (mg)	25.4 ± 2.6	22.8 ± 2.9	24.8 ± 2.1	30.1 ± 8	26.4 ± 3.6
Right adrenal (mg)	26.1 ± 5.4	21.1 ± 3.7	22.2 ± 2.9	22.5 ± 4.4	23.7 ± 2.5

Weights: Mean ± SD

*Significantly different from Control (by Dunnett's test)

Spleen: one rat from the middle-dose group and one rat from the high-dose group bearing large granular lymphocyte leukemia were excluded.

Liver:one rat from the middle-dose group and one rat from the high-dose group bearing large granular lymphocyte leukemia were excluded.

Left kidney: one rat from the middle-dose group and one rat from the high-dose group bearing kidney tumor were excluded.

Right adrenal: one rat bearing severe pheochromocytoma in the middle-dose group was excluded.

Table S5. Hematological analyses of rats at the terminal necropsy

Item		Control N=8	Low N=8	Middle N=7[1)]	High N=7[2)]
Total WBC	$(x10^2/\mu L)$	16.5±4.0	18.6±3.3	16.7±2.8	15.4±4.1
Lymphocyte	$(x10^2/\mu L)$	9.1±2.4	10.2±2.2	8.7±1.8	7.3±2.2
Neutrophil	$(x10^2/\mu L)$	6.7±1.8	7.6±1.6	7.4±1.2	7.4±2.3
Eosinophil	$(x10^2/\mu L)$	0.24±0.20	0.37±0.19	0.31±0.11	0.38±0.13
Monocyte	$(x10^2/\mu L)$	0.47±0.15	0.48±0.13	0.43±0.09	0.39±0.17
Basophil	$(x10^2/\mu L)$	0	0	0	0
RBC	$x10^4/\mu L$	906±164	778±42	839±78	847±89
HGB	(g/dL)	14.5±2.2	12.8±0.5	13.6±0.9	13.7±1.5
HCT	(%)	46.8±7.5	41.3±1.8	44.1±3.6	44.4±4.9
MCV	(fL)	51.8±1.6	53.1±1.1	52.6±1.1	52.4±0.6
MCH	(pg)	16.08±0.60	16.43±0.54	16.27±0.54	16.21±0.35
MCHC	(g/dL)	31.05±0.50	31.98±0.55	30.91±0.47	30.93±0.49
PLT	$(x10^2/\mu L)$	67.9±5.7	70.8±9.3	72.5±6.4	74.2±5.5

Mean ± SD

WBC, white blood cell count; RBC, red blood cell count; HGB, hemoglobin concentration; HCT, hematocrit level; MCV, mean corpuscular volume; MCH, mean corpuscular hemoglobin; MCHC, mean corpuscular hemoglobin concentration; PLT, platelet count

1) One rat bearing large granular lymphocyte leukemia in the middle-dose group was excluded.

2) One rat bearing large granular lymphocyte leukemia in the high-dose group was excluded.

Table S6. List of all lung tumors found in this study

Group	Time of dissection (week)	Diagnosis	Location	Size	Found at
Control	104	BAC	right caudal lobe	4 mmΦ	necropsy
Low-dose	101	BAA	right cranial lobe	3 mmΦ	necropsy
	104	BAC	left lobe	3x4 mm	necropsy
	104	BAA	right cranial lobe	2x3 mm	necropsy
Middle-dose	104	BAC	left lobe	5x6 mm	necropsy
	104	ASC	right caudal lobe	7 mmΦ	necropsy
	104	BAC	left lobe	6x7 mm	necropsy
	104 *	BAC	accesssory lobe	3x4 mm	necropsy
		BAA	left lobe	1.5x3.5 mm	trimming
	104	BAA	right middle lobe	3x4 mm	necropsy
	104	BAA	left lobe	7 mmΦ	necropsy
	104	BAC	right caudal lobe	3 mmΦ	necropsy
High-dose	96	BAA	right cranial lobe	2x2 mm	necropsy
	97	SCC	right caudal lobe	3 mmΦ	necropsy
	98	BAA	right caudal lobe	1.5x1.75 mm	histology
	104	BAA	left lobe	5 mmΦ	necropsy
	104	BAA	right middle lobe	3 mmΦ	necropsy
	104	BAC	accesssory lobe	4 mmΦ	necropsy
	104	BAC	right caudal lobe	5 mmΦ	necropsy
Ex-high-dose	101	BAA	accesssory lobe	0.5x0.75 mm	histology
	104	BAC	right caudal lobe	6x8 mm	necropsy
	104	BAA	right caudal lobe	4 mmΦ	necropsy

BAA: Bronchiolo-alveolar adenoma, BAC: Bronchiolo-alveolar adenocarcinoma, ASC: Adenosquamous carcinoma, SCC: Squamous cell carcinoma

*Two tumors were found in the same animal

Table S7. List of animals bearing proliferative lesions in the pleura in this study

Group	Time of dissection (week)	Condition	Macroscopic findings*	Histopathological classification
Middle-dose	97	Sacrificed		Mesothelioal cell hyperplasia
	104	Terminal sacrifice	numerous middle/small nodules	Mesothelioma, biphasic
	104	Terminal sacrifice	plaque-like	Mesothelioma, biphasic
	104	Terminal sacrifice		Mesothelioal cell hyperplasia
	104	Terminal sacrifice		Mesothelioal cell hyperplasia
	104	Terminal sacrifice		Mesothelioal cell hyperplasia
High-dose	51	Sacrificed		Mesothelioal cell hyperplasia
	77	Dead	numerous small nodules; hemothorax (13 mL)	Mesothelioma, epithelioid
	87	Sacrificed	plaque-like; numerous small nodules; hemothorax (15	Mesothelioma, epithelioid
	90	Sacrificed	a few large nodules; plqaue-like; numerous small nodules; hemothorax (5 mL)	Mesothelioma, sarcomatoid
	91	Sacrificed	numerous small nodules; hemothorax (4 mL); shown in Figure 3G	Mesothelioma, sarcomatoid
	94	Sacrificed	several middle nodules; numerous small nodules; plaque-like	Mesothelioma, sarcomatoid
	96	Sacrificed	plaque-like; small nodules; cardiac tamponade; hemothorax (2 mL)	Mesothelioma, sarcomatoid
	98	Sacrificed	numerous small nodules; hemothorax (10 mL)	Mesothelioma, sarcomatoid (ossification)
	99	Sacrificed	plaque-like; a large nodule; numerous small nodules	Mesothelioma, sarcomatoid
	101	Sacrificed	plaque-like; cardiac tamponade; hemothorax (15 mL)	Mesothelioma, sarcomatoid
	104	Terminal sacrifice	numerous small nodules	Mesothelioma, sarcomatoid
	104	Terminal sacrifice	plaque-like; numerous small nodules	Mesothelioma, epithelioid
	104	Terminal sacrifice		Mesothelioal cell hyperplasia
	104	Terminal sacrifice		Mesothelioal cell hyperplasia
	104	Terminal sacrifice		Mesothelioal cell hyperplasia
	104	Terminal sacrifice		Mesothelioal cell hyperplasia
Ex-High-dose	73	Sacrificed	a few large nodules; plqaue-like; several small nodules; cardiac tamponade; hemothorax (2 mL)	Mesothelioma, epithelioid
	77	Sacrificed	a few large nodules; cardiac tamponade; hemothorax (12 mL); shown in Figure 3I	Mesothelioma, sarcomatoid
	79	Sacrificed	plaque-like; hemothorax (9 mL)	Mesothelioma, sarcomatoid
	82	Sacrificed	numerous middle/small nodules	Mesothelioma, sarcomatoid (ossification)
	85	Sacrificed	numerous small nodules; hemothorax (11 mL)	Mesothelioma, sarcomatoid
	88	Sacrificed	plaque-like	Mesothelioma, sarcomatoid (ossification)
	92	Sacrificed	numerous middle/small nodules; hemothorax (10 mL); shown in Figure 3H	Mesothelioma, sarcomatoid
	97	Sacrificed	numerous small nodules; cardiac tamponade; hemothorax (2 mL)	Mesothelioma, epithelioid (keratinization)
	97	Dead	numerous middle/small nodules; cardiac tamponade; hemothorax (7 mL)	Mesothelioma, sarcomatoid (ossification)
	97	Sacrificed	plaque-like; numerous small nodules	Mesothelioma, epithelioid
	97	Sacrificed		Mesothelioal cell hyperplasia
	98	Sacrificed	plaque-like; hemothorax (1 mL)	Mesothelioma, sarcomatoid
	101	Sacrificed	plaque-like; numerous small nodules ; hemothorax (1	Mesothelioma, epithelioid (keratinization)
	102	Sacrificed	numerous small nodules; hemothorax (2 mL)	Mesothelioma, biphasic
	104	Terminal sacrifice	plaque-like	Mesothelioma, sarcomatoid

*Diameter of the tumor nodule: small, around 1 mm; middle, around 5mm; large, more than 10 mm

Table S8. Incidences of other tumors

Organ system	Neoplasm	Control N=38	Low N=39	Middle N=40	High N=40	Ex-High N=20
Hematopoietic system						
Hemolymphoid system	Large granular lymphocytic leukemia	6	6	7	6	2
	Lymphoma, malignant	1	0	0	0	0
Thymus	Lymphoma, malignant	0	1	0	1	0
Digestive system						
Tongue	Papilloma, squamous cell	1	1	0	0	0
Small intestine	Leiomyosarcoma	1	0	0	0	0
Mouth/Oral cavity/Palate	Papilloma, squamous cell	1	0	0	0	0
	Amelanotic melanoma, malignat	0	0	1	0	0
Urinary system						
Kidney	Adenoma, renal tubule	0	0	0	1	0
	Carcinoma, renal tubule	0	0	2	0	0
Urinary bladder	Carcinoma, transitional cell	1	1	0	0	0
Reproductive system						
Mammary gland	Adenocarcinoma	0	0	1	0	0
Testis	Leydig cell tumor, benign	33	31	37	34	15
	Leydig cell tumor, malignant	0	1	0	0	0
Preputial gland	Adenocarcinoma	1	0	1	1	0
	Carcinoma, squamous cell	1	0	0	0	0
Endocrine system						
Pituitary gland	Adenoma, pars distalis	7	3	2	1	3
	Ganglioneuroma, benign	0	0	0	0	1
	Carcinoma, pars distalis	1	1	0	0	0
Thyroid gland	Adenoma, C-cell	5	2	9	7	3
	Carcinoma, C-cell	1	0	2	2	0
Parathyroid gland	Adenoma	0	1	0	1	0
Adrenal gland	Pheochromocytoma, benign	3	6	4	5	2
	Carcinoma, cortical	0	0	0	0	1
	Pheochromocytoma, malignant	0	0	1	0	0
	Complex pheochromocytoma	0	0	0	0	1
Pancreatic islet	Adenoma, islet cell	8	10	8	5	3
	Carcinoma, islet cell	0	0	1	0	0
	Mixed acinar-islet cell tumor	0	2	0	0	0
Nervous system						
Brain	Oligodendroglioma, malignant	0	1	0	1	0
	Ependymoma, malignant	0	0	1	0	0
Trigeminal nerve	Schwannoma, malignant	0	0	0	1	0
Special sense system						
Ear	Neural crest tumor	0	1	0	0	0
Zymbal's gland	Carcinoma	1	0	0	0	0
Integumentary system						
Skin/Subcutaneous tissue	Fibroma	6	2	4	8	2
	Hemangiosarcoma	0	1	0	1	0
	Keratoacanthoma	1	0	0	1	0
	Schwannoma, benign	0	1	0	0	0
	trichoepithelioma	0	0	1	0	0
	Tumor, neural crest, benign	0	0	1	0	0
	Carcinoma, basal cell	0	0	0	1	0
	Schwannoma, malignant	0	0	0	1	0
Musculoskeletal system						
Muscle	Hemangiosarcoma（thigh）	0	1	0	0	0
Bone	Osteosarcoma（pelvis）	0	0	1	0	0
Abdominal cavity						
Peritonium	Mesothelioma, malignant, originated from tunica vaginalis	2	5	6	3	2
	Mesothelioma, malignant, not originated from tunica vaginalis	0	0	0	0	1
Unknown		0	0	1	0	0

Table S9. Anatomical distribution of bronchiolo-alveolar hyperplasia

	Control	Low	Middle	High
Central region	0	0	4	4
Proximal acinus region	3	5	8	7
Distal acinus region	2	4	9	7

Table S10. Mean doses for each administration (μg/rat)

	Low<br>0.0175 mg/kg	Middle<br>0.07 mg/kg	High<br>0.28 mg/kg	Ex-High<br>0.42 mg/kg
1st	4.0	16.2	65.6	98.5
2nd	4.3	17.3	68.7	103.5
3rd	4.5	17.9	71.2	107.3
4th	4.6	18.4	73.1	110.1
5th	4.8	19.0	75.5	113.9
6th	4.9	19.6	78.1	117.4
7th	5.1	20.1	79.9	119.3
8th	5.2	20.4	81.4	121.8
9th	5.3	20.7	82.6	123.9
10th	5.3	20.9	83.4	124.8
11th	5.3	21.1	84.1	123.9
12th	5.4	21.3	84.9	126.4
13th	5.5	21.6	85.7	128.3
Total doses (μg/rat)	64.2	254.5	1014.2	1518.9

The dose was set corresponding to the individual body weight of each animal for every administration.

Table S11. Comparison of the mesothelial lesions between the inhalation study and ITI studies based on the lung and pleural burdens

	Concentration/ Total Dose (µg/kg)	Total dose (µg/rat)	Lung Burden		Fibers in the PLF		Mesothelial hyperplasia		Pleural mesothelioma	
			week 13	week 104	week 13	week 104	animal number examined	animal number with lesions*	animal number examined	animal number with neoplasm
Inhalation	0 mg/m3			0 µg/lung		0	50	3	50	0
(Kasai et al., 2016)[14]	0.02 mg/m3			10.0 µg/lung		38	50	3	50	0
	0.2 mg/m3			152.4 µg/lung		134	50	7	50	0
	2 mg/m3			1,797.8 µg/lung		1,468	50	12	50	0
Current ITI	0 µg/kg	0 µg/rat	0 µg/rat	0 µg/lung	0	0	38	0	38	0
	227.5 µg/kg	64 µg/rat	30.2 µg/rat	6.2 µg/lung	24,210	20,448	36	0	39	0
	910 µg/kg	255 µg/rat	191.0 µg/rat	153.5 µg/lung	74,314	38,122	39	7	40	2
	3,640 µg/kg	1,014 µg/rat	980.4 µg/rat	922.6 µg/lung	166,528	158,563	39	15	40	11
	5,460 µg/kg	1,518.9 µg/rat	1,368.8 µg/rat	1,191.2 µg/rat	327,714	238,594	19	13	20	14
Previous intermittent ITI	0 µg/kg	0 µg/rat		0 µg/lung		0	27	0	30	0
(Hojo et al., 2022)[23]	3,250 µg/kg	1,215 µg/rat		920.5 µg/lung		156,000	26	9	29	4
	13,000 µg/kg	4,770 µg/rat		3,615.4 µg/lung		1,115,000	24	17	28	12

* numbers of animal with hyperplaisas in the visceral or parietal pleura
