# Supplementary material for: Dose–Response Relationships for Lung Tumor and Pleural Mesothelioma Induction by Repetitive Intratracheal Instillation of a Multiwalled Carbon Nanotube, MWCNT-7, in Rats: Comparison with Inhalation Carcinogenicity Based on Lung Burden
Source: Nanomaterials (Basel). 2026 Aug 20;16(16):1039. doi: 10.3390/nano16161039 (PMC13515796; doi:10.3390/nano16161039)
Supplement: Supplementary file 1 [file nanomaterials-16-01039-s001.zip › nanomaterials-4473910-supplementary-figures.pdf]

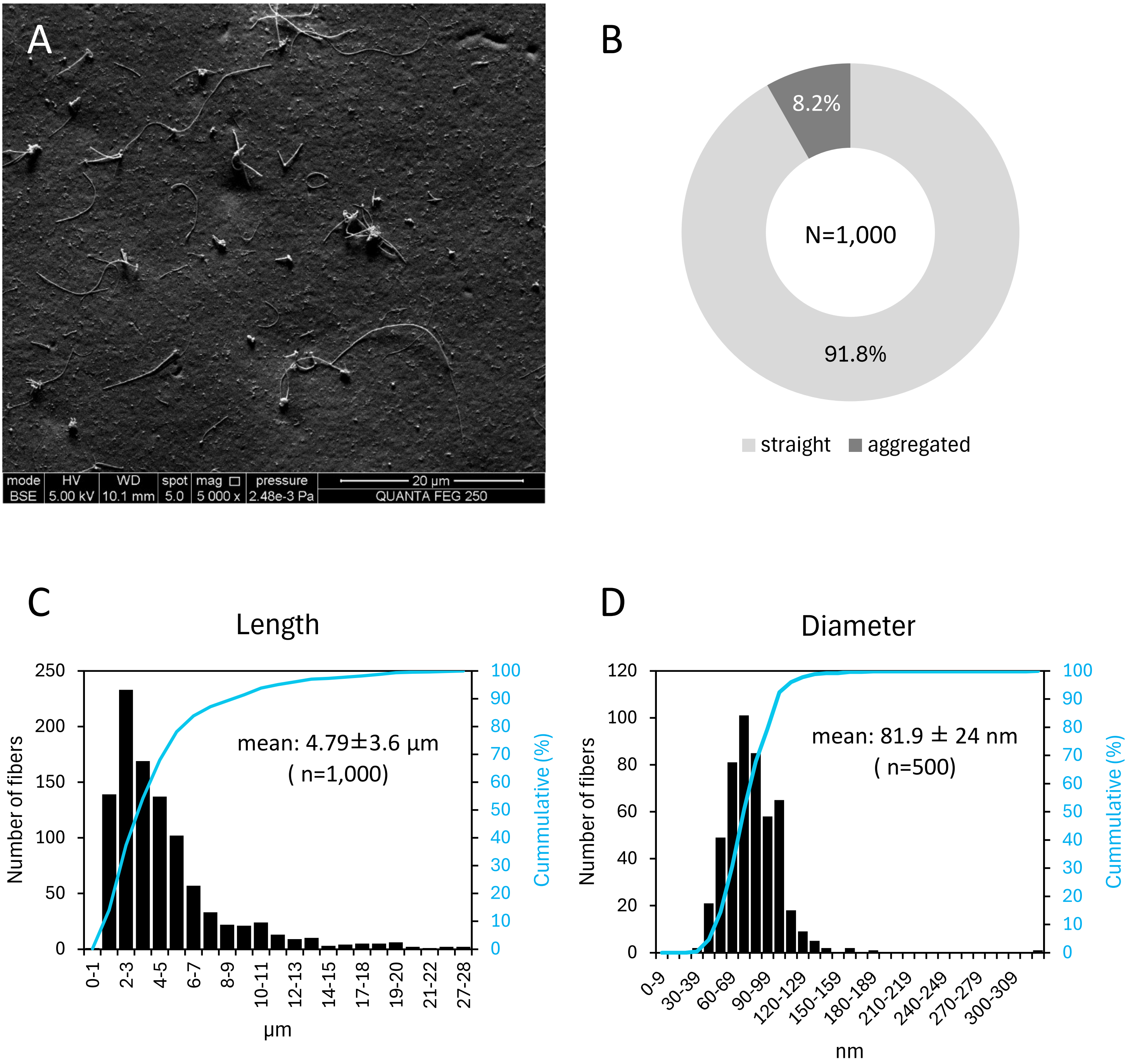

Figure S1 Characterization of bulk MWCNT-7.

(A) SEM image of bulk MWCNT-7. (B) Morphology of the individual fibers. Singular, straight-type fibers account for 91.8% of the total examined. The content of aggregated fibers (8.2%) was significantly higher than the Taquann-MWCNT-7 (see Figure 1B). (C, D) Distribution of fiber length and diameter of bulk MWCNT-7. This shows Taquann treatment changed neither the length nor diameter of the bulk MWCNT-7 (compare to Figure1C and D).

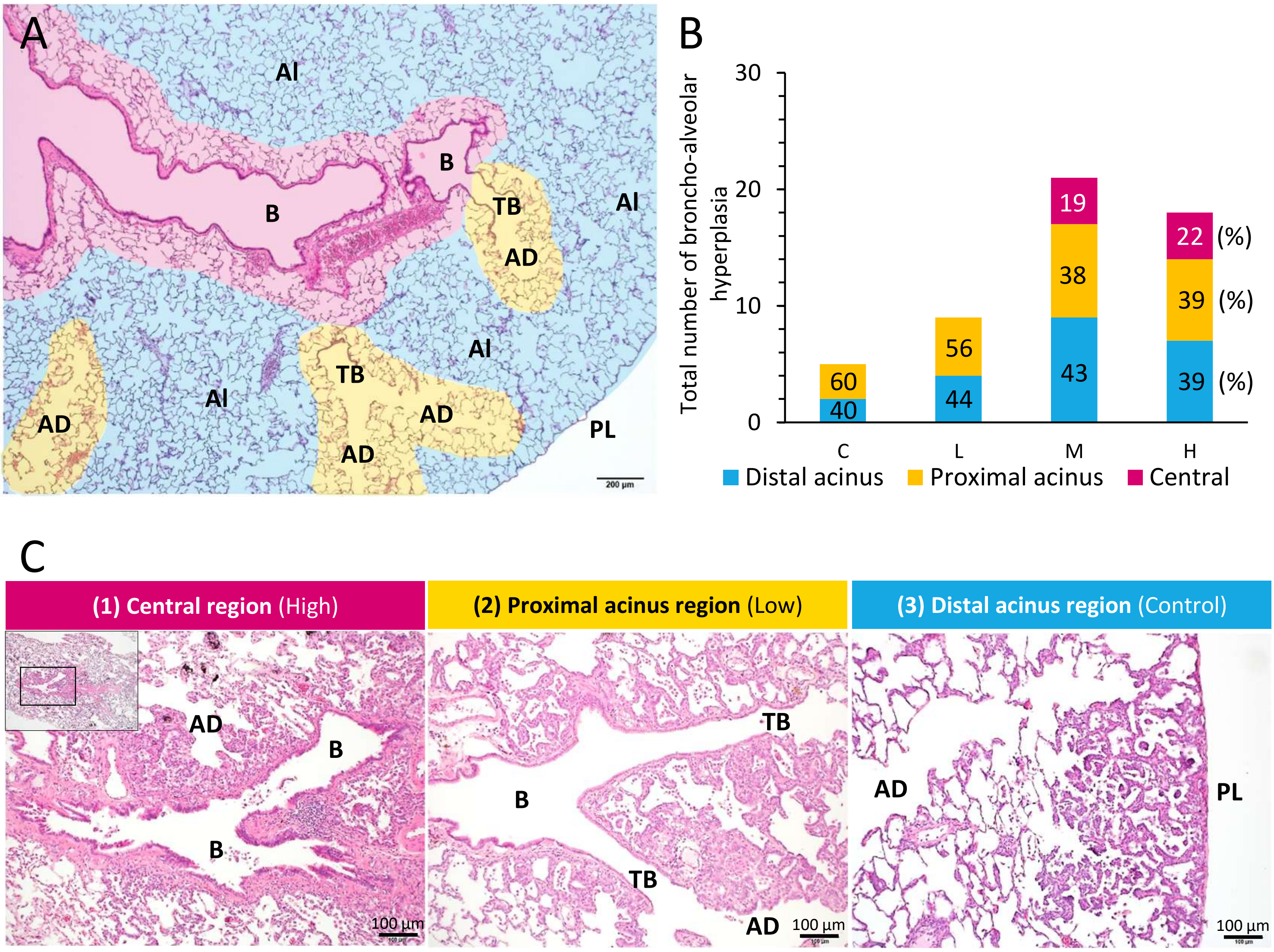

Figure S2 Histological distribution of bronchiolo-alveolar hyperplasia.

(A) Definition of the histological area. The lung was divided into three histological regions: (1) the central region, comprised of bronchi (red), (2) the proximal acinus, comprised of terminal bronchioles and alveolar ducts (yellow), and (3) the distal acinus region, comprised of all other parenchymal regions, mainly alveoli (blue). (B) The bars represent the numbers of hyperplastic lesions found in each histological region. The numbers within the bars represent percentages. The bars are the sums of the lesions found among all examined animals in each group (40 animals per group in the main 4 groups; 5 lobes per animal; see Table S1). In the control and low-dose groups, the hyperplastic lesions were observed exclusively within the acinus regions. In contrast, in the middle-dose and high-dose groups, they were detected in the acinus regions and also in the central region. (C) Representative histological images of the lesions found in each region.

B; bronchiole, TB; terminal bronchiole, AD; alveolar duct, Al; alveoli, Pl; pleura

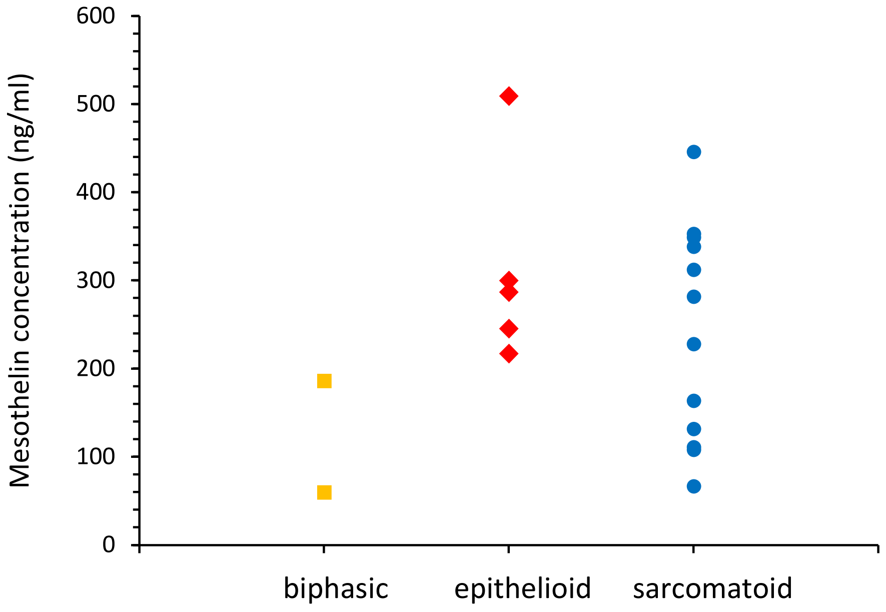

Figure S3 Comparison of the serum mesothelin levels amongst mesothelioma-bearing rats with different histological types.
The individual data shown in Figure 6D were reanalyzed based on histological diagnosis. There were no significant differences in the mesothelin concentration between different histological types.

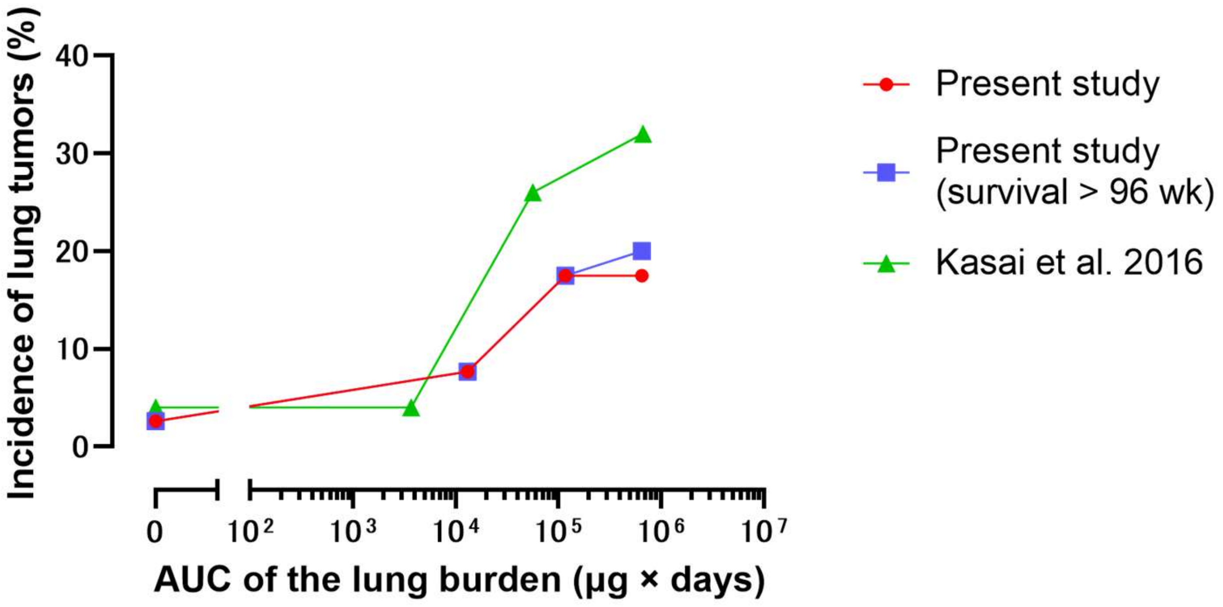

	Group	Peak lung burden (µg/rat)	AUC (µg × days)	Lung tumor (%)
Present study	Control	0	0	2.6
	Low-dose	30.2	13,093	7.7
	Middle-dose	191.0	117,704	17.5
	High-dose	980.4	650,704	17.5
Kasai et al., 2016; Kasai and Fukushima, 2023	0 mg/m^3	0	0	4
	0.02 mg/m^3	10	3,700	4
	0.2 mg/m^3	152.4	56,388	26
	2.0 mg/m^3	1797.8	665,186	32

Figure S4 Comparison of the lung tumor inducibility between the ITI study and the inhalation study based on the AUC of the lung burdens.

Dose-response curves were redrawn using the metric, area under the curve (AUC) of the lung burden (peak lung burden): duration (µg) × dose (day). The AUCs in the present study were derived from a simple geometric calculation using the same data shown in Figure 8 while those in the inhalation study were obtained from a previous study by Kasai and Fukushima, 2023 [54].

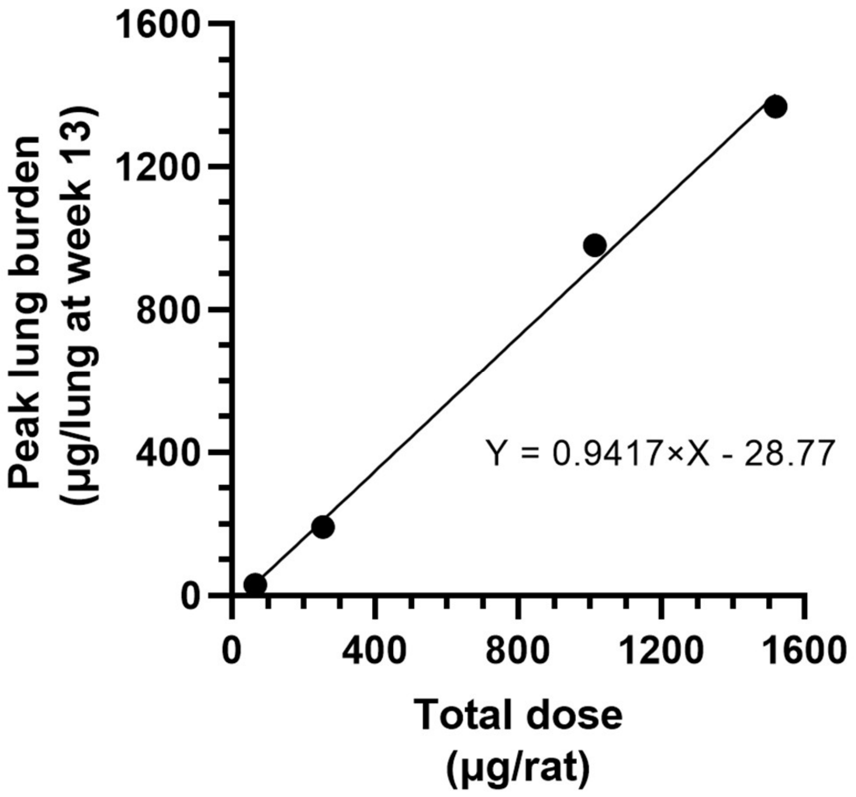

Figure S5 Correlation of the total dose and peak lung burden at week 13.
